# Computational and Crystallographic Examination of Naphthoquinone Based Diarylethene Photochromes

**DOI:** 10.3390/molecules25112630

**Published:** 2020-06-05

**Authors:** Dinesh G. Patel, Martial Boggio-Pasqua, Travis B. Mitchell, Ian M. Walton, William R. Quigley, Frank A. Novak

**Affiliations:** 1 Department of Chemistry, the Pennsylvania State University at Hazleton, Hazleton, PA 18202, USA; wrq5004@psu.edu (W.R.Q.); fxn449@gmail.com (F.A.N.); 2 Laboratoire de Chimie et Physique Quantiques, CNRS/Université Paul Sabatier, 31062 Toulouse, France; 3 Department of Chemistry, the State University of New York at Buffalo, Buffalo, NY 14260-3000, USA; travismi@buffalo.edu (T.B.M.); ian.walton@chbe.gatech.edu (I.M.W.)

**Keywords:** diarylethenes, photochromes, quinones, TD–DFT computations

## Abstract

Photochromic compounds have a lengthy history of study and a profusion of applications that stand to gain from these studies. Among the classes of photochromic compounds, diarylethenes show desirable properties including high fatigue resistance and thermal stability, thus meeting some of the most important criteria necessary to enter the realm of practical applications. Recently, photochromic diarylethenes containing quinone functionalities have demonstrated interesting optical and solid-state properties. When properly interfaced with suitable electron withdrawing groups on the aryl component, both the ring-opening and ring-closing reactions can be achieved with visible light; this is in contrast to most other diarylethenes where UV light is required for ring closure. Unfortunately, quantitative conversion from open to closed forms is not possible. In this work, we examine the relative energies of conformations of solid-state structures observed by X-ray crystallography and evaluate their thermal stabilities based on density functional theory (DFT) calculations. Time-dependent DFT (TD–DFT) is used to model the UV-vis absorption spectra of these quinone diarylethenes. We show that spectral overlap between open and closed forms is a major hindrance to full photoconversion.

## 1. Introduction

Photochromic molecules [1,2], described as reversible dyes under photochemical control [1], have proven themselves attractive as molecular scale switches with the means to optically control or gain information about systems of interest. Isomerization between two unique optical states is achieved by light of different wavelengths, though thermally induced isomerization can occur in some cases. These isomers, in addition to having different light absorption profiles, possess different electronic properties and molecular structures. Initially finding practical applications in ophthalmic lenses, specialty inks and in novelty items, potential applications for photochromic molecules have greatly increased in recent years. Taking advantages of these photonically reversible changes, researchers have demonstrated the use of photochromes in sensors [3,4,5,6,7] including dosimetery [8], magnetic switches [9,10], data storage [11,12,13,14] and photomechanical materials [15,16,17,18,19,20,21]. Even biological applications [22,23], transistor behavior [24,25,26] and digital processing [27] have been examined.

**Figure 1:**
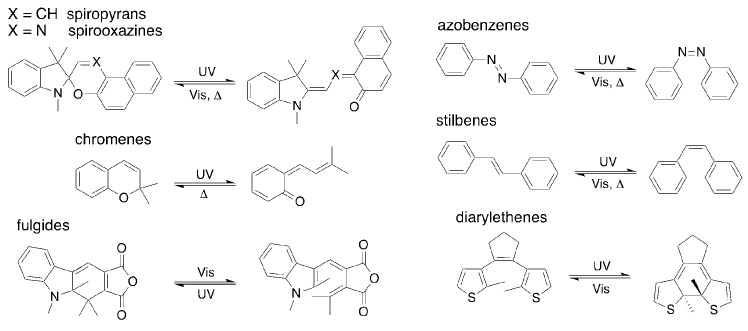
Classes of organic photochromic compounds.

The most cited classes of organic photochromes and their means of isomerization (optical and/or thermal) are shown in Figure 1. Spiropyrans, spirooxazines, azobenzenes and diarylethenes units constitute some of the most studied compounds as determined by prevalence in the literature. Among the compounds in Figure 1, diarylethenes and fulgides are notable as their thermal isomerization pathways are restricted through careful molecular design. Between the two classes, however, it can be argued that diarylethenes have received greater attention.

Diarylethene photoisomerization is typically driven by UV light for ring closure, while ring opening is facilitated by visible light (Figure 1). Given the limitations inherent to using UV-light for materials of practical utility, much research lately has been directed towards achieving photoswitching that is affected by visible light in both directions. This would increase the applicability and appeal of the diarylethene class of photochromes [28,29,30,31,32]. Quinone based diarylethene photochromes (qDAEs) have emerged as compliments to the better-known hexafluorocyclopentene based diarylethenes [33,34] and similar scaffolds. Fortuitously these quinone containing diarylethenes possess highly desirable photoisomerization driven by visible light in both the ring closing and ring opening directions [35].

Unfortunately, unlike hexafluorocyclopentene and similar diarylethenes, qDAEs have shown only 5–30% photoconversion [36] as opposed to the near quantitative conversion that can be found in other DAEs that photoswitch with UV light [1]. This inability to achieve close to 100% conversion from ring open to ring closed isomers has largely precluded a complete understanding of the electronics of the closed form. A greater understanding of the electronic and structural limitations of current qDAEs is essential to the molecular design of more effective qDAEs in the future.

**Figure 2:**
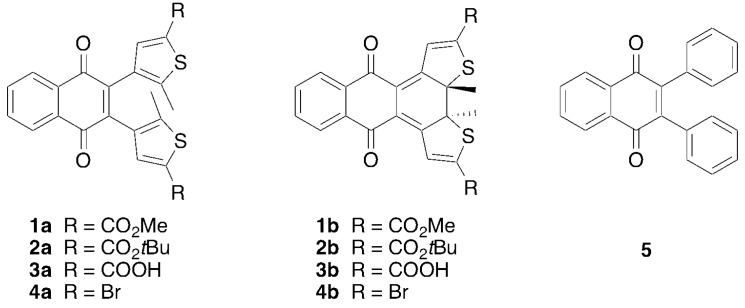
Structures of 4-naphthoquinone diarylethenes (nqDAEs) **1**–**4** in the open and closed forms as well as the structure of phenyl nqDAE **5**.

**Scheme 1:**
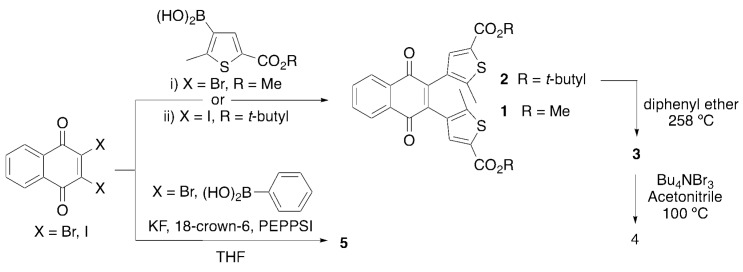
Synthetic routes to the compounds in this study. Reagents used: (i) CsF, PEPPSI-IPr, dimethoxyethane, (ii) K_3_PO_4_, Pd(OAc)_2_, H_2_O/dioxane.

DAEs have been the subject of many theoretical studies [37] aimed at understanding their thermal biostability and optical properties but also their photoswitching and photodegradations mechanisms, highlighting the critical role of extended conical intersection seams [38,39]. To our knowledge, only one report exists of the computational analysis of a qDAE [40]. Herein we examine the structural and electronic restrictions of qDAEs that lead to the observed reduced photoconversion. This study involves a series of photochromic 2,3-bisthienyl-1,4-naphthoquinone diarylethenes (nqDAEs, Figure 2). The selected thiophenes are diester substituted (compounds **1** and **2**), dicarboxylic acid substituted (compound **3**) or dibromide substituted (compound **4**), all of which have some degree of electron withdrawing character present on the molecule. Bisphenyl substituted 1,4-naphthoquinone compound **5** was also synthesized and has recently been reported [41] by a different synthetic route than reported here (Scheme 1). These diarylethene molecules were selected partly because the electronic structure of the quinone-DAE system results in a donor-acceptor system [42] that effectively lowers the electronic energy gap into the range of visible light.

## 2. Results and Discussion

### 2.1. Synthesis and Characterization

The realization of functionally diverse photochromic qDAEs has proven a synthetic challenge in that Stille coupling reactions have, until recently, been exclusively employed to couple halogenated quinones to stanyllated thiophenes [36,40,43,44]. Harsh conditions are required to install the necessary trialkyl tin coupling partner, and this concomitantly restricts molecules to possessing only functional groups that can tolerate the organolithium promoted stannylation step. As the variety of functional groups is limited, so are the potential applications of qDAEs. The use of milder Suzuki methods, for example transition metal catalyzed installation of boronic acids and esters without the need for organolithium reagents [45,46], allows the inclusion of nucleophile sensitive functional groups.

We have previously reported the synthesis and characterization data of compounds **1**–**4** [35]. Briefly, in our original synthesis 2,3-dibromo-1,4-naphthoquinone is coupled, via a Suzuki reaction, to the boronic acid of 5-methyl-2-thiophene carboxylic acid methyl ester or the analogous t-butyl ester (Scheme 1). Loss of the t-butyl group and subsequent bromination of the resulting acid lead to compounds **3** and **4**, respectively. As we have noted, this reaction does not appear to proceed under the most common Suzuki conditions. Anhydrous conditions were employed to prevent the naphthoquinone coupling partner from decomposing under aqueous conditions [47,48]. To synthesize compound **5**, similar conditions were used with the commercially available phenyl boronic acid. During the preparation of this manuscript, compound **5** was reported by Migulin [41], who synthesized and used the more reactive 2,3-diiodo-1,4-naphthoquinone with a traditional aqueous Suzuki coupling scheme. We have used this as the basis for our synthesis of **2** in this study.

Purification of **1**–**5** is achieved by column chromatography using a hexanes/ethyl acetate gradient eluent system. Curiously, the ring-closed forms of **1** and **2** have higher R_f_ values, thus irradiating (405–410 nm) the impure compound band immediately after beginning chromatography leads to a green band that is easily isolable. By using this technique, we isolated a miniscule amount of pure closed form **1b** with crystals grown by slow evaporation of the chromatographed fractions. The crystals attained were not enough for nuclear magnetic resonance spectroscopy (NMR) or UV-Vis experiments but it was possible to select a single crystal and an X-ray structure was acquired.

### 2.2. Optical Studies and Photochromic Properties

**Figure 3:**
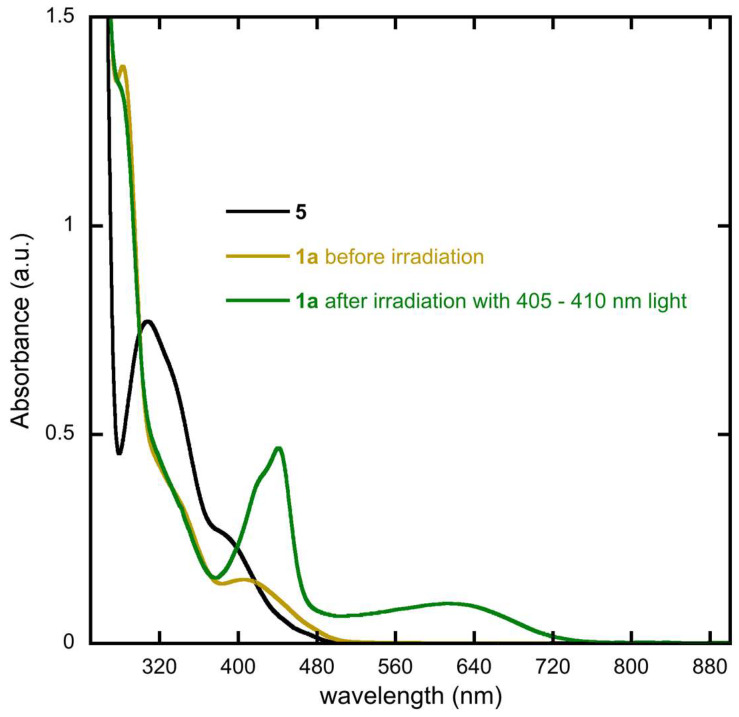
Experimental UV-Vis spectra of pure **1a**, a solution of **1a** after irradiation with 405–410 nm wavelength light and **5** in dichloromethane. Concentrations are on the order of 10^−5^ M. The solution of **1a** after irradiation contains a mixture of **1a** and **1b.**

Within the context of quinone based diarylethenes, Deng and Liebeskind [36] reported that complete conversion between open and closed forms is not possible. The reason for this inability to quantitatively photoisomerize was not discussed, nor has its origins been probed since. We, too, report the same observation with methyl ester substituted **1** as evidenced by irradiation of an ^1^H NMR sample [35]. The UV-Vis spectra of **1** and **5** in dichloromethane (DCM) are presented in Figure 3. Despite being synthesized previously, the UV-Vis spectrum of both compounds has not been examined in DCM. As expected, compound **5** is not photochromic but compound **1** shows new peaks at 612 nm and 440 nm following irradiation with 405–410 nm light.

### 2.3. X-Ray Crystallography

In our initial attempts at understanding the behavior of these photochromes we turned to structural analysis through single crystal X-ray diffraction techniques. We found it difficult to grow crystals of most of our compounds, though fortuitously, crystals of **1b** and **5** are easily obtained by slow evaporation of solvent from hexane/ethyl acetate chromatographic fractions. Crystals of **1b** were grown with the exclusion of light. Efforts at growing crystals of **1a** and **2a** gave amorphous solids; attainment of crystals of **4** has not yet been attempted.

#### 2.3.1. Compound **5**

**Figure 4:**
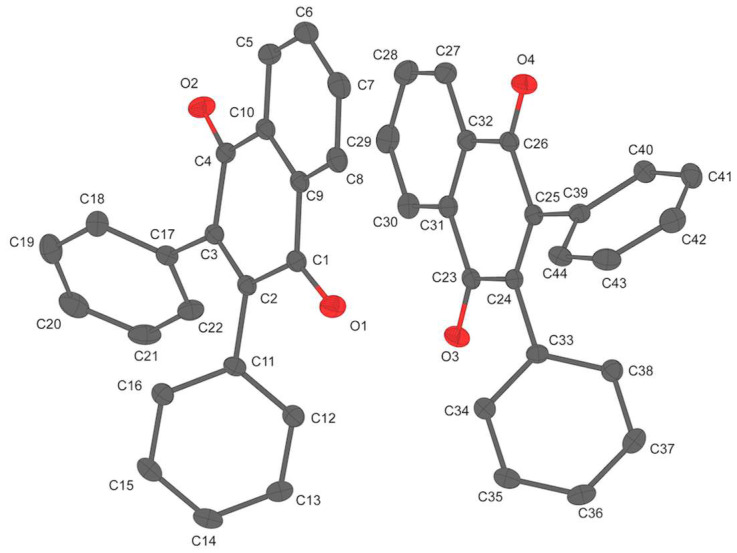
Molecular structure of the phenyl nqDAE **5** asymmetric unit with thermal ellipsoids drawn at the 50% probability level. Hydrogen atoms are omitted for clarity.

Yellow prism-shaped crystals of **5** crystallize in the centrosymmetric space group $P\bar{1}$ with an asymmetric unit comprising two crystallographically unique phenyl nqDAE molecules (Figure 4). Both unique molecules in **5** assume the anti-parallel conformation with active C—C separation distances of 3.209(2) Å (C16···C22) and 3.256(2) Å (C38···C44). The two unique molecules form dimers through a slipped π-π stacking mode in which both the quinoid (Cg1, yellow) and benzenoid ring (Cg2, magenta) of one unique molecule interact with the benzenoid ring (Cg4, cyan) of the other unique molecule (Supporting Information, Figure S5). Additionally, the dimers are stabilized by two sets of hydrogen bonding (Supporting Information, Figure S5) between a quinoid oxygen atom of one molecule and a phenyl hydrogen atom of the other molecule and an electrostatic intreaction between a quinoidal oxygen on one molecule and a carbonyl carbon of the other molecule (C23···O1=3.128(1) Å) [49]. Further, the dimers interact with neighboring dimers through an extensive hydrogen-bonding network, which acts as a stabilizing force for the lattice (Supporting Information, Figure S7 and Table S3). Finally, it is worth mentioning that the quinoid ring Cg4 is non-planar while the quinoid ring Cg1 is planar. While interesting, the nature and consequences of this observation is beyond the scope of this paper.

#### 2.3.2. Closed form **1b**

**Figure 5:**
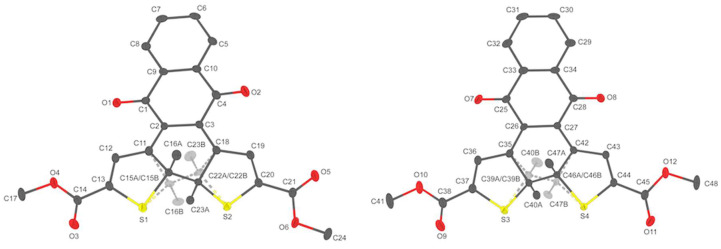
Molecular structure of the two crystallographically unique molecules of phenyl nqDAE **5** with thermal ellipsoids drawn at the 50% probability level. Hydrogen atoms are omitted for clarity. Transparent dashed lines and atoms represent modeled disorder. Gray: Carbon, Red: Oxygen; Yellow: Sulfur.

The closed nqDAE isomer **1b**, gained by brief irradiation during column chromatography, was crystallized by slow evaporation of a hexanes/ethyl acetate chromatographic fraction. The resulting dark green crystals were found to be suitable for analysis by single-crystal X-ray diffraction (SCXRD). Compound **1b** crystallizes in the P2_1_/c space group (crystallographic details are provided in the Supporting Information). Analysis of the crystal structure found that there were two crystallographically distinct molecules in the asymmetric unit (Figure 5). The two distinct molecules stack with overlap of the quinone backbone and one of the thiophene methyl ester groups. The stacking of the aromatic portion of the quinone backbone is similar to the classic π-π slip stacking model [50,51]. The phenyl rings of the naphthoquinone backbones have a centroid to centroid distance of 3.5517(12) Å with a shift of 1.210(3) Å. Additionally, the distance from the quinone centroid to the phenyl centroid is 3.708(11) Å with a 1.610(3) Å offset. Orientation of the two crystallographically distinct naphthylquinone backbones position one of the quinone oxygens 3.3437(16) Å from the centroid of the other quinone ring. The specific interactions are shown in the Supporting Information, Figure S6. When viewed down the a-axis, the role of the quinone π-π slip stacking becomes more apparent (Figure 5).

Each stacked column of quinone-DAE runs parallel to the a-axis, however the plane of the quinone backbone does not lie parallel to the b-c plane (Supporting Information, Figure S6). Each column interacts with neighboring columns through the overlap of exterior methyl ester groups. Each neighboring column has the opposite tilt of the planar backbone from the b-c plane. The angle of the quinone backbone within the stack and the position of the neighboring quinone results in a slip stacking of the phenyl region. Aromatic slip stacking has been found to be a preferred energetic minimum and is often present in crystal structures of aromatic molecules [50,51]. Interestingly there is also an overlap of carbonyl groups of the methyl ester groups between stacks. The orientation of the overlap is suggestive of some level of π-π interaction and electrostatic interactions between opposing carbonyl carbons and oxygens, though further studies would be needed to confirm specific stabilizing interactions.

### 2.4. Computations

#### 2.4.1. Conformational Analysis and Thermal Stability of Compound **1**

**Figure 6:**
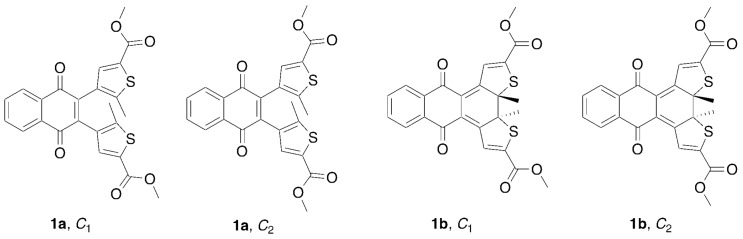
Conformations for compound **1** with symmetry given.

X-ray analysis of **1b** (Figure 5) shows two distinct isomers present in the crystal structure. We label these isomers based on their symmetry (Figure 6). The conformers, **C_1_-closed** and **C_2_-closed** differ only in the relative orientations of the methyl ester groups due to free rotation about the thiophene-ester bond. In **C_1_-closed** the ester carbonyl groups adopt a “transoid” type geometry while in **C_2_-closed** these groups adopt a “cisoid” type geometry. Given the ratio of **C_1_-closed** and **C_2_-closed** in the crystal structure, we surmise that these two forms are close in energy. As part of our computational analysis, however, we also examined the analogous open form conformers **C_1_-open** and **C_2_-open**. Other potential conformers were not examined.

Geometry optimizations of open- and closed-ring isomers in the gas phase and in DCM were performed using density functional theory (DFT). The geometry optimization results obtained for examining the various conformers is presented in Table 1. All energies are relative to ***C*_1_-open** (**1a**, *C*_1_). From the data, it is evident that the conformers ***C*_1_-open** (**1a**, *C*_1_) and ***C*_2_-open** (**1a**, *C*_2_) are near identical in energy with differences of 0.8 kJ·mol^−1^ and 0.4 kJ·mol^−1^ in the gas phase and in DCM, respectively. When examining closed forms ***C*_1_-closed** (**1b**, *C*_1_) and ***C*_2_-closed** (**1b**, *C*_2_), we see differences of 0.3 kJ·mol^−1^ both in the gas phase and in DCM. Considering that the ***C*_1_** and ***C*_2_** conformers are quasi-isoenergetic, we will only discuss results on the ***C*_1_** conformers of compound **1** in the following.

The energy difference between the open and closed form is estimated at 44 kJ·mol^−1^ and 50 kJ·mol^−1^ in the gas phase and in DCM, respectively. The activation energy for the cyclization is calculated in DCM at 174 kJ·mol^−1^ while that for the reverse reaction is found to be 124 kJ·mol^−1^. These results indicate that compound **1** should exhibit good thermal bistability.

#### 2.4.2. Computed Spectra and Molecular Orbital Analysis for Compound **1**

**Figure 7:**
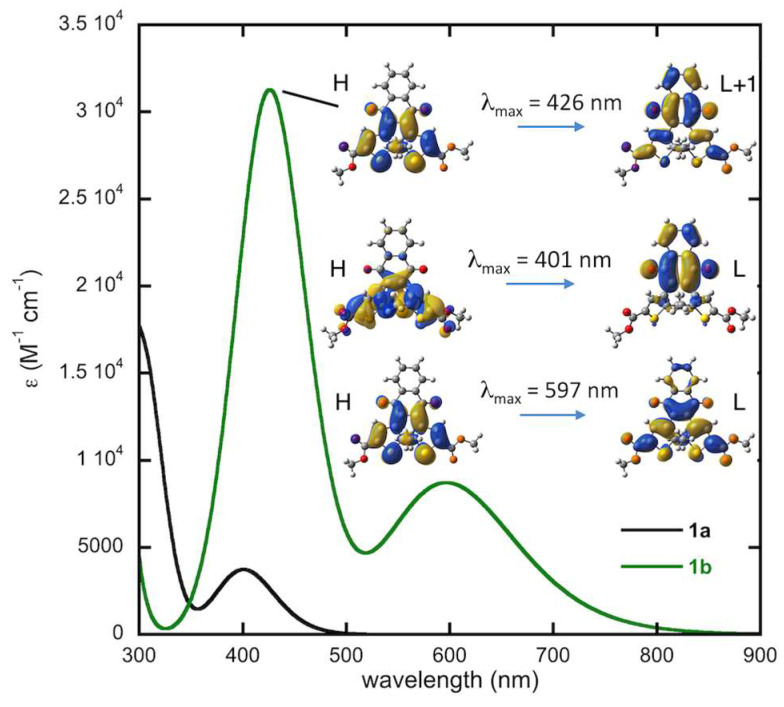
Computed spectrum of **1a** and **1b** in dichloromethane at time-dependent density functional theory (TD–DFT) level and molecular orbitals (H: HOMO, L: LUMO, isocontour: 0.02) involved in the main electronic transitions.

Figure 7 shows the computed UV-vis absorption spectra for the C_1_ conformer of **1a** and **1b** at the TD-DFT level along with the molecular orbitals involved in the main electronic transitions. The spectrum of **1a** has the correct line shape compared to the experimental spectrum (Figure 3) with the first maximum predicted at 401 nm, close to the experimental value of 406 nm. While the experimental spectrum for the pure closed-form **1b** was not obtained, as the photostationary state is a mixture of **1a** and **1b**, the computed spectrum of **1b** is consistent with the line shape of the experimental spectrum shown in Figure 3. The simulated spectrum presents two maxima at 597 and 426 nm, in good agreement with the two absorption bands appearing at 612 nm and 441 nm upon irradiation of **1a**. Note that the absorption maxima are slightly blue-shifted in the calculated spectra but that this shift (<0.2 eV) is well within the typical margin of error for such methods.

#### 2.4.3. Modeling of the Ratio of Open and Closed Forms of **1b**

Experimentally, upon irradiation we see a new absorption band appear in the region of 600 nm, attributed to the closed form **1b**. In our previous experiments [35], we irradiated dilute solutions (~10^−5^ M) for 20 s with 405–410 nm light to reach the photostationary state. We then turned to ^1^H NMR spectroscopy to obtain the ratio of **1a**:**1b** after irradiation. Given that the NMR data [35] showed only ~22% photoconversion, using that number we modeled the expected spectra in this work. Further, for this work we examined various irradiation times and find that the photostationary state, using our setup [35], is reached upon 10 s. In the supporting information, video files of concentrated solutions are provided illustrating these conversions. Given the higher sample concentrations needed for easily observed color changes, however, longer and arbitrary irradiation times were used.

**Figure 8:**
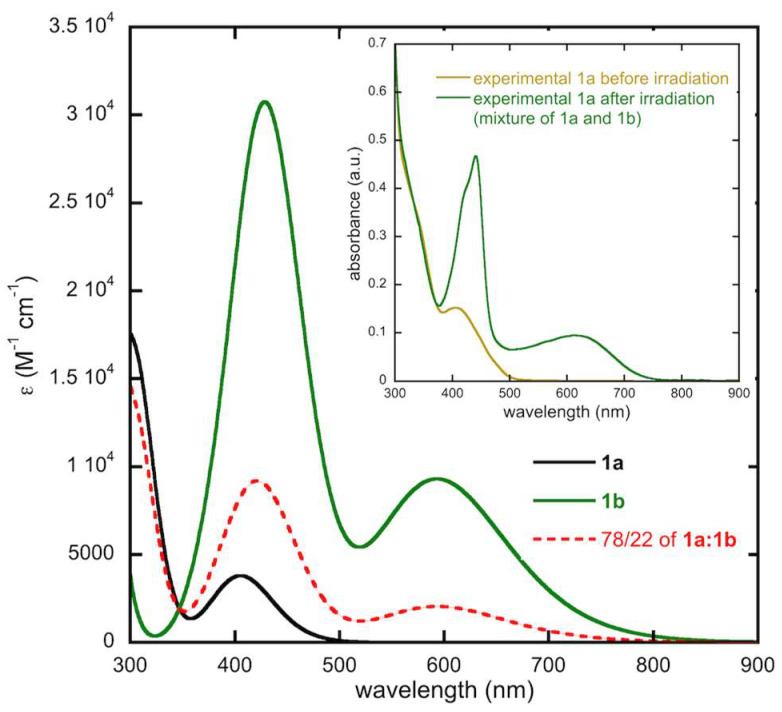
Computed spectrum of the photostationary state obtained upon irradiation of **1a** in dichloromethane at TD-DFT level, along with the computed spectrum of **1a** and pure **1b** given as a reference. The photostationary state assumes a 78/22 ratio of **1a**:**1b**. The inset shows the obtained experimental data.

In order to model the spectrum of the photostationary state observed experimentally, we have simulated the absorption spectrum of a mixture made of 78% of **1a** and 22% of **1b**, as deduced from NMR data [35]. The result is shown in Figure 8 in comparison with the experimental spectrum (inset). A very nice agreement is obtained with the appearance of a broad band centered around 600 nm and the rise of sharper and more intense band in the 400 nm region.

#### 2.4.4. Thermal Stability, Computed Spectra and Molecular Orbital Analysis for Compound **3**

The dicarboxylic acid nqDAE **3** presents similar ground-state energetic properties as the diester derivatives **1** and **2**. The closed-ring isomer **3b** is computed at 48 kJ·mol^−1^ and 54 kJ·mol^−1^ above the open-ring isomer **3a** in the gas phase and in DCM, respectively. The energy barriers for the cyclization and cycloreversion reactions are 174 kJ·mol^−1^ and 120 kJ·mol^−1^ in DCM, respectively. These results are similar to the diester nqDAEs within a few kJ·mol^−1^, indicating similar thermal stabilities this series of compounds.

**Figure 9:**
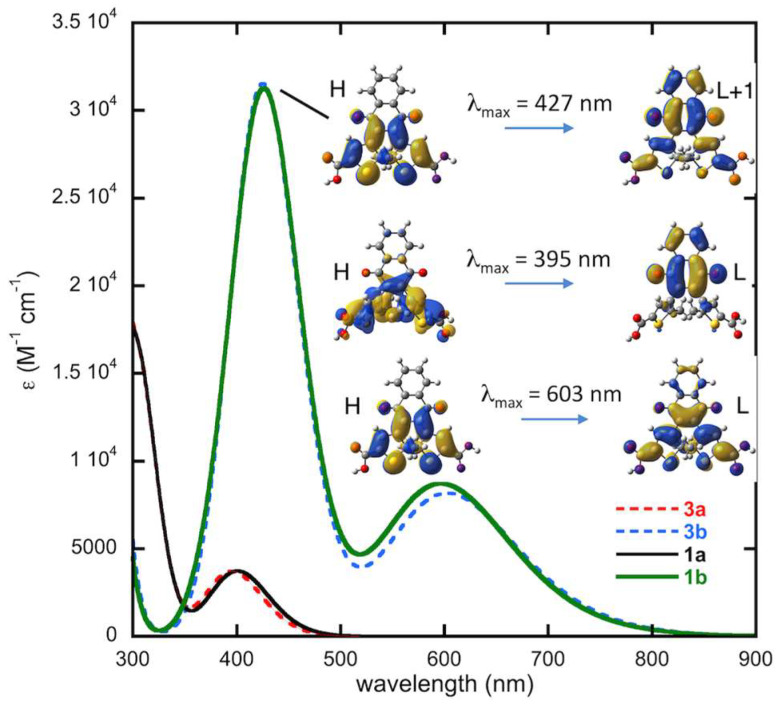
Computed spectrum of **3a** and **3b** in dichloromethane at TD-DFT level and molecular orbitals (H: HOMO, L: LUMO, isocontour: 0.02) involved in the main electronic transitions. The computed spectra for **1a** and **1b** are shown for comparison.

Figure 9 illustrates the comparison between the simulated UV-vis absorption spectra of the open and closed forms of compounds **1** and **3**. It clearly appears that the acid derivative displays very similar spectra to the methyl ester derivative with both the band positions and intensities being unchanged. We thus also expect a similar photochromic behavior for the dicarboxylic acid nqDAE compared to the diester nqDAEs.

#### 2.4.5. Conformational Analysis and Thermal Stability of Compound **4**

Bromine qDAE compound **4** presents only the C_2_ conformer for the open and closed forms due to symmetry. Unlike the diester and dicarboxylic acid nqDAEs, which present very similar thermal stabilities and optical properties, the bromide nqDAE **4** presents some differences with these derivatives. First, the thermal stability is predicted to be improved for compound **4** by the calculations. The closed-ring isomer **4a** is found at a very similar energy to the open-ring form, only 5 kJ·mol^−1^ and 12 kJ·mol^−1^ above it in the gas phase and in DCM, respectively. This is a drastic reduction of the energy gap between these two isomers compared to the diester and dicarboxylic acid nqDAEs. In addition, while the energy barrier for the cyclization computed at 173 kJ·mol^−1^ remains similar to derivatives **1** – **3**, that of the cycloreversion is largely increased as it is computed at 161 kJ·mol^−1^, suggesting an improved thermal bistability for the bromide nqDAE.

#### 2.4.6. Computed Spectra and Molecular Orbital Analysis for Compound **4**

**Figure 10:**
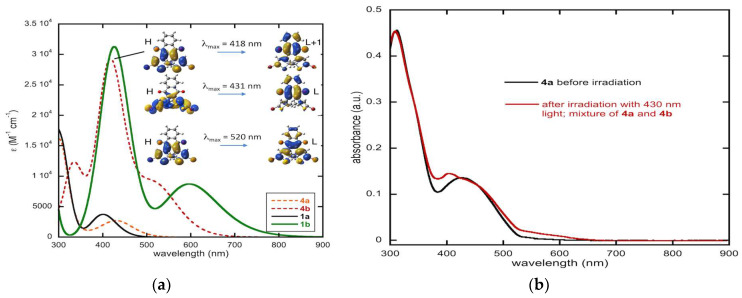
(**a**) Computed spectrum of **4a** and **4b** in dichloromethane at TD-DFT level and molecular orbitals (H: HOMO, L: LUMO, isocontour: 0.02) involved in the main electronic transitions. The computed spectra of **1a** and **1b** are shown for comparison. (**b**) Experimental spectrum of **4a** and the spectrum obtained after irradiation of **4a** with 430 nm wavelength light.

The calculated and experimental optical properties of compound **4** are illustrated in Figure 10. Figure 10a shows the comparison between the simulated UV-Vis absorption spectra of **1** with **4**. Substantial differences between **1** and **4** can be observed. On the one hand, the absorption band of the open-ring isomer **4a** in the 400 nm region is substantially red shifted compared to **1a**. The maximum of this absorption band is computed at 431 nm compared to 401 nm in **1a**. This represents a bathochromic shift of 1736 cm^–1^. On the other hand, the absorption of the closed-ring isomer **4b** becomes blue shifted compared to **1b**. In particular, the maximum of the first absorption band is moved from 597 nm to 520 nm, corresponding to a hypsochromic shift of 2480 cm^–1^. The intensities of these bands are comparable between compounds **4** and **1**. Note, however, that despite these substantial shifts in the absorption, there is still a strong overlap of the first absorption band of the open-ring isomer **4a** with the second absorption band of the more intense closed-ring isomer **4b** in the 400 nm region. This is probably the main reason for the inefficient photocyclization reaction in these nqDAEs.

Experimentally, this absorption band shifts from 427 nm before irradiation to 404 nm after irradiation (Figure 10b). In the previous report of irradiation of **4**, 405–410 nm light was used. Here, we employed a 430 nm diode as the irradiation source to better overlap with the absorption band of **4a**; however, we do not see an increase in the long wavelength absorption band due to the closed form.

## 3. Materials and Methods

### 3.1. General

Experimental UV-Vis spectra were obtained in dichloromethane (DCM) for **1**, **2**, **4** and **5** or acetone for **3** using a Shimadzu UV-1800 spectrometer. Irradiation was achieved using a home-built light source [35]. All irradiation experiments were conducted using a 405–410 nm light emitting diode with the exception of the irradiation of **4**, which was conducted using a 430 nm light emitting diode. NMR spectra were recorded on a Bruker AVIII-500 spectrometer operating at 500 MHz for ^1^H and 125 MHZ for ^13^C. As this data has been previously presented for **1**–**4** [35] and **5 [41]**, it is not included here.

Tetrahydrofuran (THF) and dioxane were dried over sodium/benzophenone and stored over 4 Å molecular sieves. All other reagents, including palladium catalyst PEPPSI-IPr (CAS Number: 905459-27-0), were obtained from commercial suppliers and used as received. Flash column chromatography was accomplished using silica gel obtained from Fisher Scientific (230–400 mesh).

### 3.2. Synthesis

The syntheses of **1**, **3** and **4** have been previously been reported and the molecules characterized by ^1^H NMR, ^13^C NMR and high resolution mass spectrometry. Our synthetic routes and characterization matched that in the literature [35].

Given the new synthetic approach by Migulin [41] we opted to synthesize compound **2** through the use 2,3-diiodo-1,4-naphthoquinone under aqueous conditions. Briefly, (5-(tert-Butoxycarbonyl)-2-methylthiophen-3-yl)boronic acid (1.949 g, 8.05 mmol), 2,3-diiodo1,4-naphthoquinone (1.500 g, 3.66 mmol), potassium phosphate (4.660 g, 21.95 g), water (15 mL), dioxane (45 mL) and palladium acetate (0.016 g, 0.07 mmol) were added to a 100 mL Schlenk flask. The mixture was degassed by three freeze-pump-thaw cycles, then backfilled with nitrogen and allowed to stir until starting the quinone and monocoupled product were no longer visible by thin layer chromatography (silica plate, 15% ethyl acetate in hexanes). The reaction was then diluted with ethyl acetate (150 mL) and washed with water (3 × 50 mL) and brine (50 mL). The organic phase was dried over magnesium sulfate, then adsorbed onto silica. The crude product was chromatographed using a silica column eluting with 100% hexanes to 20% acetone in hexanes. The pure fractions were collected affording a yellow solid upon removal of solvent. The yellow solid slowly turned dark green upon exposure to room light (1.280 g, 64%). Characterization data matched that of compound **2** obtained by our previous synthetic route [35]. While the yield was comparable to that reported using anhydrous Suzuki methodology, the chromatography was much cleaner.

Compound **5**. In a 100 mL Schlenk tube was placed KF·2H_2_O (1.788 g, 18.99 mmol) followed by heating to ~120 °C for 30 min while under high vacuum. Upon cooling and backfilling with nitrogen, 2,3-dibromo-1,4-naphthoquinone (1.000 g, 3.17 mmol), phenylboronic acid (1.158 g, 9.50 mmol), 18-crown-6 ether (0084 g, 0.32 mmol), PEPPSI (0.043 g, 0.06 mmol) and anhydrous THF (15 mL) were added under a stream of nitrogen. The yellow reaction mixture was subjected to three freeze-pump-thaw cycles, backfilled with nitrogen and refluxed overnight (15 h). The yellow-brown mixture obtained was diluted with ethyl acetate (100 mL) and washed with water (3 × 20 mL) and brine (20 mL). The organic phase was dried over magnesium sulfate, filtered and solvent removed under reduced pressure to give a yellow solid. The solid was adsorbed onto silica and chromatographed on silica eluting with 100% hexanes to 9:1 hexanes/ethyl acetate. Upon standing, some crystals formed and were saved for X-ray crystallographic analysis. The remainder of the product was collected by removal of solvent to yield a yellow solid (0.549 g, 56%). Characterization data matched that in the literature [41].

### 3.3. Computational

DFT has been used to perform geometry optimizations of the nqDAEs **1**–**4** in their ground electronic state. These calculations were performed in the gas phase and in dichloromethane using the polarizable continuum model (PCM). The optimizations were carried out for the open- and closed-ring isomers of these compounds and for the transition states connecting them. Harmonic frequency analysis was performed on the optimized structures to verify the nature of the stationary points (minima or transition states) on the potential energy surface. These transition states correspond to open-shell singlet (diradical) species and were computed using broken-symmetry DFT calculations. To account for the spin contamination, spin-projected energies were computed with an approximate spin-correction procedure proposed by Yamaguchi and coworkers [52,53]. All these geometry optimizations were performed with the B3LYP functional [54] and the 6-31G(d) basis set [55,56].

The UV-vis absorption spectra were simulated at the TD-DFT level using the long-range corrected CAM-B3LYP functional [57] along with the 6-311+G(d,p) basis set [58,59]. The use of a range-separated functional was necessary in order to describe the electronic transitions to charge transfer states accurately. All absorption spectra were computed using PCM to describe the dichloromethane solvent The convoluted spectra were obtained using a phenomenological Gaussian broadening characterized by a half-width at half-height of 2000 cm^–1^ for each vertical transition. The effect of the functional and basis set on these spectra is presented in Supporting Information.

All the calculations were performed with the Gaussian 09 series of programs [60].

### 3.4. X-ray Crystallographic Data Collection and Refinement

Parameters for the crystal structures of **1b** and **5** are shown in the supporting information (Table S1).

Single crystals of **1b** and **5** suitable for X-ray diffraction were mounted on the tip of a glass fiber using Paratone oil under atmospheric conditions and placed on a Bruker SMART APEX II CCD diffractometer installed on a rotating anode source (Mo-Kα radiation, λ = 0.71073 Å) with a detector distance of 40.00 mm from the crystal. During the data collection, the crystals were cooled to 90(1) K using an Oxford Cryosystem (Cryostream 700) nitrogen gas-flow apparatus. Frames were collected for **1b** using two 180° ω-scans (0.5° scan width) at different φ-angles (φ = 0 to 72° at 72° increments) and stopped halfway through run two for a total of 540 frames, nominally covering complete reciprocal space. Frames were collected for **5** using the same data acquisition strategy except that five 180 ω-scans (0.5 scan width) were used with φ = 0 to 288° at 72° increments for a total of 1800 frames, nominally covering complete reciprocal space. Data reduction was completed using SAINT version 8.40A and a multi-scan absorption correction was applied using SADABS version 2016 included in the Bruker APEX3 software suite [61]. Space-group determination was performed using XPREP utility included in the SHELXTL software package [62]. The structures were solved with ShelXT [63] using intrinsic phasing and refined with ShelXL [64] using least-squares minimization (full-matrix least-squares on F^2^). Non-hydrogen atoms were refined anisotropically and hydrogen atoms were placed at calculated idealized position and refined using a riding model.

Disorder of the cyclized thiophene rings for **1b** was resolved through the free refinement of the disordered atoms (bridging thiophene carbons and inner methyl groups), with the corresponding atoms split between major and minor location. Free refinement of the disordered atoms suggests a 70/30 ratio between molecular orientation in each of the distinct orientations in each crystallographically distinct molecule. Examination of the packing shows why the disorder between the two orientations of the thiophenes does not disrupt the packing. The methyl groups reside in nearly the exact same location, regardless of orientation and the difference in the bridging thiophene carbon is less than 1 Å.

## 4. Conclusions

Previously, qDAE and nqDAE closed forms were obtained only upon treatment of open forms with strong Lewis acids [36,40]. Here we have shown that closed forms can be obtained by irradiation of an impure sample during chromatography with the closed form eluting faster than the corresponding open form. Specifically, this allowed for the isolation of **1b**, giving single crystals upon slow evaporation of solvent and facilitating a greater investigation of the ring-closed isomer. Crystals of **1b** and **5** were obtained and examined by single crystal X-ray diffraction. To our knowledge, this is the first report of a closed form qDAE crystal structure. To obtain **1** and **5** in this study, we employed anhydrous Suzuki conditions; using Migulin’s newly reported synthesis of 2,3,-diiodonaphthoquinone [41] and employing this highly reactive compound, we were able to obtain **2** under typical Suzuki coupling conditions.

Compounds **1**–**4** were subjected to computational analysis at the TD-CAM-B3LYP/6-311+G(d,p) level of theory. The simulated UV-vis absorption spectra closely match the experimental spectral data, most notably for systems containing a mix of ring open and ring closed isomers and suggest that these computations would be useful for modeling other similar systems that achieve a photostationary equilibrium in solution. Importantly, the relatively low photoswitching efficiency of these nqDAEs compared to other DAEs is explained by the overlap of absorption bands between the open and closed forms leading to incomplete conversion from the open- to the closed-ring isomer. Additionally, the obtained data suggests a high thermal biostability for these compounds. The thermal stability and unique electronic structures of quinone DAEs make this class of DAE a very promising subclass of photochromes.

## Figures and Tables

**Table 1:** Relative Conformer Energies for compound 1.

Symmetry	Geometry	ΔE (gas Φ) kJ/mol	ΔE (in DCM) kJ/mol
***C***_1_	Open	0.0	0.0
***C***_2_	Open	0.8	0.4
***C***_1_	Closed	43.6	49.9
***C***_2_	Closed	43.9	50.2

## Supplementary Materials

Figure S1: Computed UV-Vis spectra of the conformers for open form isomer **1a** in DCM and in the gas phase at TD-B3LYP/6-31G*, Figure S2: Computed UV-Vis spectra of the conformers for closed form isomer **1b** in dichloromethane and in the gas phase at TD-B3LYP/6-31G* level, Figure S3: Computed spectrum of the *C*_1_ conformers of **1a** and **1b** in DCM at TD-CAM-B3LYP/6-31G* level, Figure S4: A comparison of TD-CAM-B3LYP/6-31G* and TD-CAM-B3LYP/6-311+G** simulated spectra for the *C*_1_ conformers of **1a** and **1b**, Figure S5: Computed UV-Vis spectra of **2a** and **2b** in dichloromethane, Figure S6: ORTEP of phenyl nqDAE **5** illustrating the dimer formation through π-π stacking, hydrogen bonding and electrostatic interactions, Figure S7: Interactions of the naphthoquinone backbone facilitating the stacking observed in the crystal structure of **1b**, Figure S8: Extensive hydrogen-bonding network of **5** viewed down the crystallographic a-axis, Figure S9: Comparison of the planar and non-planar naphthoquinone regions of compound **5**, Table S1: Crystal data and structure refinement for Compound **5** and Compound **1b**, Table S2: π-πinteractions in the lattice of Compound **5** and Compound **1b**, Table S3: Hydrogen Bond Geometry for **5**, Tables S4–S17: Optimized B3LYP/6-31G* Cartesian coordinates for compounds in this study, Video S1: Photoisomerization of **1a** to **1b**, Video S2: Photoisomerization of **1b** to **1a**. CCDC 2002586 and 2002587 contain the supplementary crystallographic data in CIF format and this data can be obtained free of charge at http://www.ccdc.cam.ac.uk/structures.
