# Supplementary material for: Computational and Crystallographic Examination of Naphthoquinone Based Diarylethene Photochromes
Source: Molecules. 2020 Jun 5;25(11):2630. doi: 10.3390/molecules25112630 (PMC7321381; doi:10.3390/molecules25112630)

checkCIF/PLATON report

Structure factors have been supplied for datablock(s) I

THIS REPORT IS FOR GUIDANCE ONLY. IF USED AS PART OF A REVIEW PROCEDURE FOR PUBLICATION, IT SHOULD NOT REPLACE THE EXPERTISE OF AN EXPERIENCED CRYSTALLOGRAPHIC REFEREE.

No syntax errors found.   CIF dictionary   Interpreting this report

Datablock: I

```
Bond precision:  C-C = 0.0026 A          Wavelength=0.71073

Cell:            a=7.1500(3)     b=25.5032(9)      c=22.9477(8)
                 alpha=90        beta=93.571(1)     gamma=90
Temperature:     90 K

                Calculated                Reported
Volume          4176.3(3)                 4176.3(3)
Space group     P 21/c                    P 1 21/c 1
Hall group      -P 2ybc                   -P 2ybc
Moiety formula  C24 H18 O6 S2             1(C24 H18 O6 S2)
Sum formula     C24 H18 O6 S2             C24 H18 O6 S2
Mr              466.50                    466.50
Dx,g cm-3       1.484                     1.484
Z               8                         8
Mu (mm-1)       0.296                     0.296
F000            1936.0                    1936.0
F000'           1938.94
h,k,lmax        10,36,32                  10,36,32
Nref            12798                     12747
Tmin,Tmax       0.982,0.988               0.691,0.746
Tmin'           0.943

Correction method= # Reported T Limits: Tmin=0.691 Tmax=0.746
AbsCorr = MULTI-SCAN

Data completeness= 0.996          Theta(max)= 30.554

R(reflections)= 0.0506( 9186)     wR2(reflections)= 0.1272( 12747)

S = 1.047                  Npar= 663
```

The following ALERTS were generated. Each ALERT has the format
test-name_ALERT_alert-type_alert-level.
Click on the hyperlinks for more details of the test.

Alert level C

```
PLAT094_ALERT_2_C Ratio of Maximum / Minimum Residual Density ....       2.14 Report
PLAT213_ALERT_2_C Atom O2              has ADP max/min Ratio .....        3.1 prolat
PLAT220_ALERT_2_C NonSolvent Resd 2  C   Ueq(max) / Ueq(min) Range        3.1 Ratio
PLAT250_ALERT_2_C Large U3/U1 Ratio for Average U(i,j) Tensor ....        2.2 Note
PLAT906_ALERT_3_C Large K Value in the Analysis of Variance ......      4.445 Check
PLAT911_ALERT_3_C Missing FCF Refl Between Thmin & STh/L=    0.600          8 Report
```

Alert level G

```
PLAT042_ALERT_1_G Calc. and Reported MoietyFormula Strings  Differ     Please Check
PLAT301_ALERT_3_G Main Residue  Disorder .............(Resd  1  )        13% Note
PLAT301_ALERT_3_G Main Residue  Disorder .............(Resd  2  )        13% Note
PLAT793_ALERT_4_G Model has Chirality at C15A        (Centro SPGR)          R Verify
PLAT793_ALERT_4_G Model has Chirality at C22A        (Centro SPGR)          R Verify
PLAT793_ALERT_4_G Model has Chirality at C39A        (Centro SPGR)          S Verify
PLAT793_ALERT_4_G Model has Chirality at C46A        (Centro SPGR)          S Verify
PLAT793_ALERT_4_G Model has Chirality at C15B        (Centro SPGR)          S Verify
PLAT793_ALERT_4_G Model has Chirality at C22B        (Centro SPGR)          S Verify
PLAT793_ALERT_4_G Model has Chirality at C39B        (Centro SPGR)          R Verify
PLAT793_ALERT_4_G Model has Chirality at C46B        (Centro SPGR)          R Verify
PLAT910_ALERT_3_G Missing # of FCF Reflection(s) Below Theta(Min).          1 Note
PLAT912_ALERT_4_G Missing # of FCF Reflections Above STh/L=  0.600         43 Note
PLAT941_ALERT_3_G Average HKL Measurement Multiplicity ...........        2.2 Low
PLAT978_ALERT_2_G Number C-C Bonds with Positive Residual Density.         19 Info
PLAT992_ALERT_5_G Repd & Actual _reflns_number_gt Values Differ by          1 Check
```

```
   0 ALERT level A = Most likely a serious problem - resolve or explain
   0 ALERT level B = A potentially serious problem, consider carefully
   6 ALERT level C = Check. Ensure it is not caused by an omission or oversight
  16 ALERT level G = General information/check it is not something unexpected

   1 ALERT type 1 CIF construction/syntax error, inconsistent or missing data
   5 ALERT type 2 Indicator that the structure model may be wrong or deficient
   6 ALERT type 3 Indicator that the structure quality may be low
   9 ALERT type 4 Improvement, methodology, query or suggestion
   1 ALERT type 5 Informative message, check
```

checkCIF publication errors

Alert level A

```
PUBL004_ALERT_1_A  The contact author's name and address are missing,
            _publ_contact_author_name and _publ_contact_author_address.
PUBL005_ALERT_1_A  _publ_contact_author_email, _publ_contact_author_fax and
            _publ_contact_author_phone are all missing.
            At least one of these should be present.
PUBL006_ALERT_1_A  _publ_requested_journal is missing
            e.g. 'Acta Crystallographica Section C'
PUBL008_ALERT_1_A  _publ_section_title is missing. Title of paper.
PUBL009_ALERT_1_A  _publ_author_name is missing. List of author(s) name(s).
PUBL010_ALERT_1_A  _publ_author_address is missing. Author(s) address(es).
PUBL012_ALERT_1_A  _publ_section_abstract is missing.
            Abstract of paper in English.
```

```
  7 ALERT level A = Data missing that is essential or data in wrong format
  0 ALERT level G = General alerts. Data that may be required is missing
```

Publication of your CIF

You should attempt to resolve as many as possible of the alerts in all categories. Often the minor alerts point to easily fixed oversights, errors and omissions in your CIF or refinement strategy, so attention to these fine details can be worthwhile. In order to resolve some of the more serious problems it may be necessary to carry out additional measurements or structure refinements. However, the nature of your study may justify the reported deviations from journal submission requirements and the more serious of these should be commented upon in the discussion or experimental section of a paper or in the "special_details" fields of the CIF. *checkCIF* was carefully designed to identify outliers and unusual parameters, but every test has its limitations and alerts that are not important in a particular case may appear. Conversely, the absence of alerts does not guarantee there are no aspects of the results needing attention. It is up to the individual to critically assess their own results and, if necessary, seek expert advice.

If level A alerts remain, which you believe to be justified deviations, and you intend to submit this CIF for publication in a journal, you should additionally insert an explanation in your CIF using the Validation Reply Form (VRF) below. This will allow your explanation to be considered as part of the review process.

Validation response form

Please find below a validation response form (VRF) that can be filled in and pasted into your CIF.

```
# start Validation Reply Form
_vrf_PUBL004_GLOBAL
;
PROBLEM: The contact author’s name and address are missing,
RESPONSE: ...
;
_vrf_PUBL005_GLOBAL
;
PROBLEM: _publ_contact_author_email, _publ_contact_author_fax and
RESPONSE: ...
;
_vrf_PUBL006_GLOBAL
;
PROBLEM: _publ_requested_journal is missing
RESPONSE: ...
;
_vrf_PUBL008_GLOBAL
;
PROBLEM: _publ_section_title is missing. Title of paper.
RESPONSE: ...
;
_vrf_PUBL009_GLOBAL
;
PROBLEM: _publ_author_name is missing. List of author(s) name(s).
RESPONSE: ...
;
_vrf_PUBL010_GLOBAL
;
PROBLEM: _publ_author_address is missing. Author(s) address(es).
```

```
RESPONSE: ...
;
_vrf_PUBL012_GLOBAL
;
PROBLEM: _publ_section_abstract is missing.
RESPONSE: ...
;
# end Validation Reply Form
```

If you wish to submit your CIF for publication in Acta Crystallographica Section C or E, you should upload your CIF via the web. If you wish to submit your CIF for publication in IUCrData you should upload your CIF via the web. If your CIF is to form part of a submission to another IUCr journal, you will be asked, either during electronic submission or by the Co-editor handling your paper, to upload your CIF via our web site.

PLATON version of 22/04/2020; check.def file version of 09/03/2020

Datablock I - ellipsoid plot

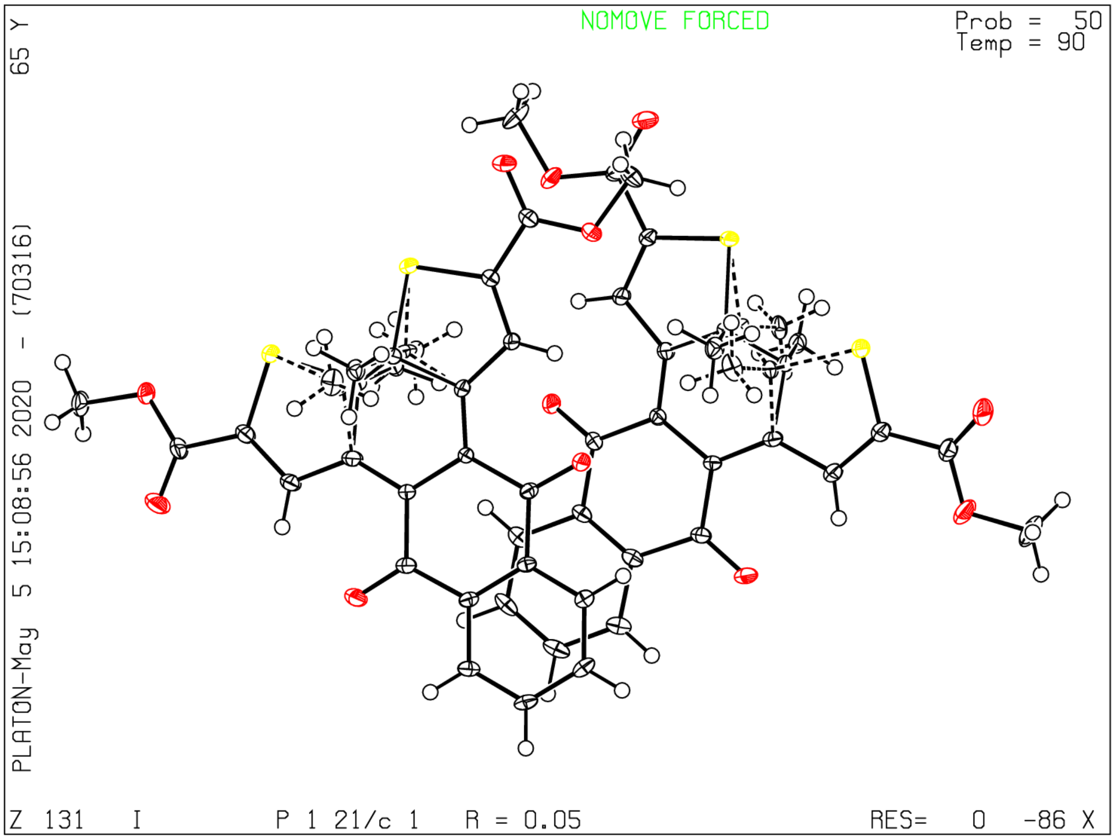

Supplement: Supplementary file 1 [file molecules-25-02630-s001.zip › Supporting Information and Figures/Crystallography Files/Compound_1b_cifreport.pdf]
