# Supplementary material for: Computational and Crystallographic Examination of Naphthoquinone Based Diarylethene Photochromes
Source: Molecules. 2020 Jun 5;25(11):2630. doi: 10.3390/molecules25112630 (PMC7321381; doi:10.3390/molecules25112630)

checkCIF/PLATON report

Structure factors have been supplied for datablock(s) compound_5

THIS REPORT IS FOR GUIDANCE ONLY. IF USED AS PART OF A REVIEW PROCEDURE FOR PUBLICATION, IT SHOULD NOT REPLACE THE EXPERTISE OF AN EXPERIENCED CRYSTALLOGRAPHIC REFEREE.

No syntax errors found.   CIF dictionary   Interpreting this report

Datablock: compound_5

```
Bond precision:  C-C = 0.0018 A          Wavelength=0.71073

Cell:          a=11.1148(6)      b=11.2447(6)     c=15.3720(8)
               alpha=70.357(1)   beta=70.476(1)   gamma=64.508(1)
Temperature:   90 K

                Calculated                 Reported
Volume          1592.17(15)                1592.17(15)
Space group     P -1                       P -1
Hall group      -P 1                       -P 1
Moiety formula  C22 H14 O2                 C22 H14 O2
Sum formula     C22 H14 O2                 C22 H14 O2
Mr              310.33                     310.33
Dx,g cm-3       1.295                      1.295
Z               4                          4
Mu (mm-1)       0.082                      0.082
F000            648.0                      648.0
F000'           648.30
h,k,lmax        14,14,20                   14,14,20
Nref            7877                       7875
Tmin,Tmax       0.992,0.995                0.707,0.747
Tmin'           0.992

Correction method= # Reported T Limits: Tmin=0.707 Tmax=0.747
AbsCorr = MULTI-SCAN

Data completeness= 1.000          Theta(max)= 28.281

R(reflections)= 0.0395( 6757)     wR2(reflections)= 0.1050( 7875)

S = 1.025                  Npar= 433
```

The following ALERTS were generated. Each ALERT has the format
test-name_ALERT_alert-type_alert-level.
Click on the hyperlinks for more details of the test.

Alert level C

```
PLAT911_ALERT_3_C Missing FCF Refl Between Thmin & STh/L=    0.600          3 Report
```

Alert level G

```
PLAT154_ALERT_1_G The s.u.'s on the Cell Angles are Equal ..(Note)      0.001 Degree
PLAT333_ALERT_2_G Large Aver C6-Ring C-C Dist C23      -C31      .       1.46 Ang.
PLAT941_ALERT_3_G Average HKL Measurement Multiplicity ...........        4.1 Low
PLAT978_ALERT_2_G Number C-C Bonds with Positive Residual Density.         25 Info
```

```
   0 ALERT level A = Most likely a serious problem - resolve or explain
   0 ALERT level B = A potentially serious problem, consider carefully
   1 ALERT level C = Check. Ensure it is not caused by an omission or oversight
   4 ALERT level G = General information/check it is not something unexpected

   1 ALERT type 1 CIF construction/syntax error, inconsistent or missing data
   2 ALERT type 2 Indicator that the structure model may be wrong or deficient
   2 ALERT type 3 Indicator that the structure quality may be low
   0 ALERT type 4 Improvement, methodology, query or suggestion
   0 ALERT type 5 Informative message, check
```

It is advisable to attempt to resolve as many as possible of the alerts in all categories. Often the minor alerts point to easily fixed oversights, errors and omissions in your CIF or refinement strategy, so attention to these fine details can be worthwhile. In order to resolve some of the more serious problems it may be necessary to carry out additional measurements or structure refinements. However, the purpose of your study may justify the reported deviations and the more serious of these should normally be commented upon in the discussion or experimental section of a paper or in the "special_details" fields of the CIF. checkCIF was carefully designed to identify outliers and unusual parameters, but every test has its limitations and alerts that are not important in a particular case may appear. Conversely, the absence of alerts does not guarantee there are no aspects of the results needing attention. It is up to the individual to critically assess their own results and, if necessary, seek expert advice.

Publication of your CIF in IUCr journals

A basic structural check has been run on your CIF. These basic checks will be run on all CIFs submitted for publication in IUCr journals (*Acta Crystallographica*, *Journal of Applied Crystallography*, *Journal of Synchrotron Radiation*); however, if you intend to submit to *Acta Crystallographica Section C* or *E* or *IUCrData*, you should make sure that full publication checks are run on the final version of your CIF prior to submission.

Publication of your CIF in other journals

Please refer to the *Notes for Authors* of the relevant journal for any special instructions relating to CIF submission.

PLATON version of 22/04/2020; check.def file version of 09/03/2020

Datablock compound_5 - ellipsoid plot

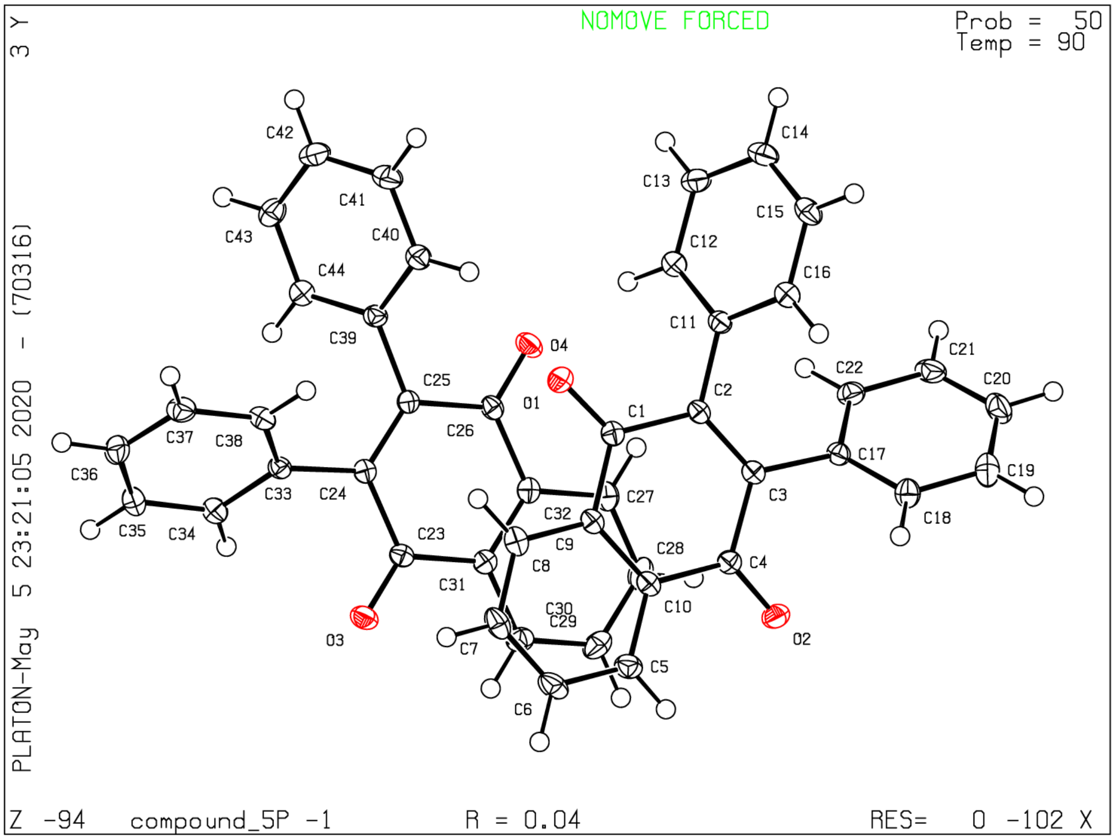

Supplement: Supplementary file 1 [file molecules-25-02630-s001.zip › Supporting Information and Figures/Crystallography Files/Compound_5_cifreport.pdf]
