# Supplementary material for: Computational and Crystallographic Examination of Naphthoquinone Based Diarylethene Photochromes
Source: Molecules. 2020 Jun 5;25(11):2630. doi: 10.3390/molecules25112630 (PMC7321381; doi:10.3390/molecules25112630)
Supplement: Supplementary file 1 [file molecules-25-02630-s001.zip › Supporting Information and Figures/molecules-812556supp pdf.pdf]

Supporting Information

Computational and Crystallographic Examination of Naphthoquinone Based Diarylethene Photochromes

Dinesh G. Patel [1,*], Martial Boggio-Pasqua [2,*], Travis B. Mitchell[3], Ian M. Walton[3], William R. Quigley[1], and Frank A. Novak[1]

[1] Department of Chemistry, the Pennsylvania State University at Hazleton, Hazleton, Pennsylvania 18202, United States
[2] Laboratoire de Chimie et Physique Quantiques, CNRS/Université Paul Sabatier, Toulouse, France
[3] Department of Chemistry, the State University of New York at Buffalo, Buffalo, New York, 14260-3000, United States

* Correspondence: dgp15@psu.edu, martial.boggio@irsamc.ups-tlse.fr

Table of Contents

Choice of functional and basis set and effect of the ester alkyl group 4

Figure S1. Computed UV-Vis spectra of the conformers for open form isomer 1a in DCM and in the gas phase at TD-B3LYP/6-31G* level and molecular orbitals involved in the main electronic transitions. 4

Figure S2. Computed UV-Vis spectra of the conformers for closed form isomer 1b in dichloromethane and in the gas phase at TD-B3LYP/6-31G* level and molecular orbitals involved in the main electronic transitions. 5

Figure S3. Computed spectrum of the C_1 conformers of 1a and 1b in DCM at TD-CAM-B3LYP/6-31G* level and molecular orbitals involved in the main electronic transitions. 6

Figure S4. A comparison of TD-CAM-B3LYP/6-31G* and TD-CAM-B3LYP/6-311+G** simulated spectra for the C_1 conformers of 1a and 1b. The respective transitions with the 6-311+G** basis set are shown. 7

Figure S5. Computed spectrum of 2a and 2b in dichloromethane at TD-DFT level. 8

Crystallography Data 9

Table S1. Crystal data and structure refinement for Compound_5 and Compound_1b 10

Figure S6. ORTEP of phenyl nqDAE 5 illustrating the dimer formation through π-π stacking, hydrogen bonding, and electrostatic interactions.. 11

Figure S7. Interactions of the naphthoquinone backbone facilitating the stacking observed in the crystal structure of 1b with centroid-to-centroid distance and oxygen to centroid distances illustrated. 12

Table S2. π-πinteractions in the lattice of Compound_5 and Compound_1b 13

Figure S8. Extensive hydrogen-bonding network of 5 viewed down the crystallographic *a*-axis. 13

Table S3. Hydrogen Bond Geometry for 5 (Å, °) 13

Figure S9. Comparison of the non-planar (top) and planar (bottom) molecules present in the crystal structure of compound 5. Planarity refers to the naphthoquinone portion of the molecules. 14

Cartesian Coordinates for Computations 15

Table S4. Optimized B3LYP/6-31G* Cartesian coordinates (in Å) for C_1 conformer of compound 1a in dichloromethane. 16

Table S5. Optimized B3LYP/6-31G* Cartesian coordinates (in Å) for C_2 conformer of compound 1a in dichloromethane. 18

Table S6. Optimized B3LYP/6-31G* Cartesian coordinates (in Å) for C_1 conformer of compound 1b in dichloromethane. 20

Table S7. Optimized B3LYP/6-31G* Cartesian coordinates (in Å) for C_2 conformer of compound 1b in dichloromethane. **22**

Table S8. Optimized broken-symmetry B3LYP/6-31G* Cartesian coordinates (in Å) for transition state between 1a and 1b in dichloromethane. **24**

Table S9. Optimized B3LYP/6-31G* Cartesian coordinates (in Å) for C_1 conformer of compound 2a in dichloromethane. **26**

Table S10. Optimized B3LYP/6-31G* Cartesian coordinates (in Å) for C_1 conformer of compound 2b in dichloromethane. **28**

Table S11. Optimized broken-symmetry B3LYP/6-31G* Cartesian coordinates (in Å) for transition state between 2a and 2b in dichloromethane. **30**

Table S12. Optimized B3LYP/6-31G* Cartesian coordinates (in Å) for C_1 conformer of compound 3a in dichloromethane. **32**

Table S13. Optimized B3LYP/6-31G* Cartesian coordinates (in Å) for C_1 conformer of compound 3b in dichloromethane. **34**

Table S14. Optimized broken-symmetry B3LYP/6-31G* Cartesian coordinates (in Å) for transition state between 3a and 3b in dichloromethane. **36**

Table S15. Optimized B3LYP/6-31G* Cartesian coordinates (in Å) for C_2 conformer of compound 4a in dichloromethane. **38**

Table S16. Optimized B3LYP/6-31G* Cartesian coordinates (in Å) for C_2 conformer of compound 4b in dichloromethane. **39**

Table S17. Optimized broken-symmetry B3LYP/6-31G* Cartesian coordinates (in Å) for transition state between 4a and 4b in dichloromethane. **40**

References **41**

Choice of functional and basis set and effect of the ester alkyl group

While not discussed in the main text, below we describe the comparison of the UV-vis absorption spectra computed at the TD-DFT level using two different functionals (B3LYP and CAM-B3LYP) and two different basis sets (6-31G(d) and 6-311+G(d,p) denoted 6-31G* and 6-311+G** hereafter).

To probe any potential differences in their absorption profiles, the conformers shown in Figure 6 (main text) were first examined by TD-DFT using the B3LYP hybrid functional and 6-31G* basis set. The modeled spectra are shown in Figure S1 for the C_1 and C_2 conformers of **1a** in the gas phase and in dichloromethane (DCM). While the line shapes match, it is found that the absorption maxima, regardless of dielectric constant, are red-shifted relative to that observed experimentally for **1a** in Figure 3. Experimentally in DCM, **1a** shows its long wavelength absorption maximum at 406 nm while TD-DFT predicts a maximum of 500 nm. This red-shift (lower energy prediction) is due to the presence of charge transfer states in the open form.

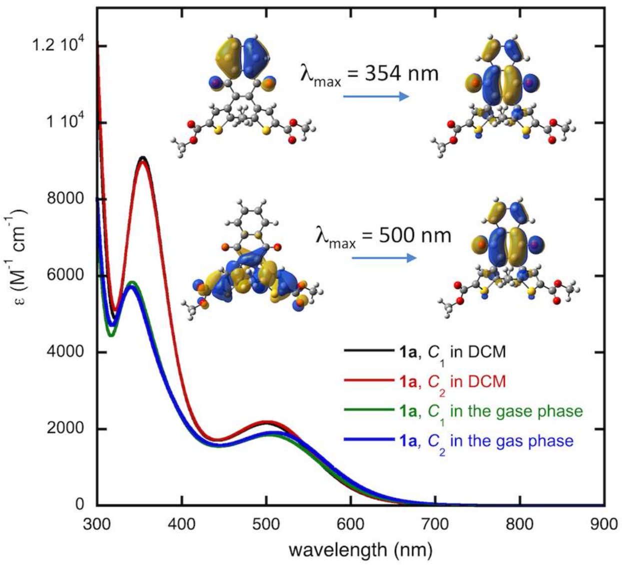

Figure S1. Computed UV-Vis spectra of the conformers for open form isomer 1a in DCM and in the gas phase at TD-B3LYP/6-31G* level and molecular orbitals involved in the main electronic transitions.

While experimental spectra for the pure closed form isomers of **1** and **2** have not been obtained, they were nonetheless modeled using the same criteria as were used for the open forms. This data is shown in Figure S2. As was observed for the open form, the spectra are all shifted to lower energies, regardless of symmetry, relative to the experimental data of

open/closed mixtures (see Figure 3 in the main text). Of particular note is the long wavelength absorption band which is centered at 612 nm in spectroscopy experiments but is shifted by 40 nm to 652 nm in the TD-B3LYP spectrum. Additionally, this longwavelength band stretches far into the near infrared with an onset past 900 nm while the experimental data shows an onset of approximately 760 nm. To better model the spectrum, the use of a range-separated funtional, CAM-B3LYP, was next investigated.

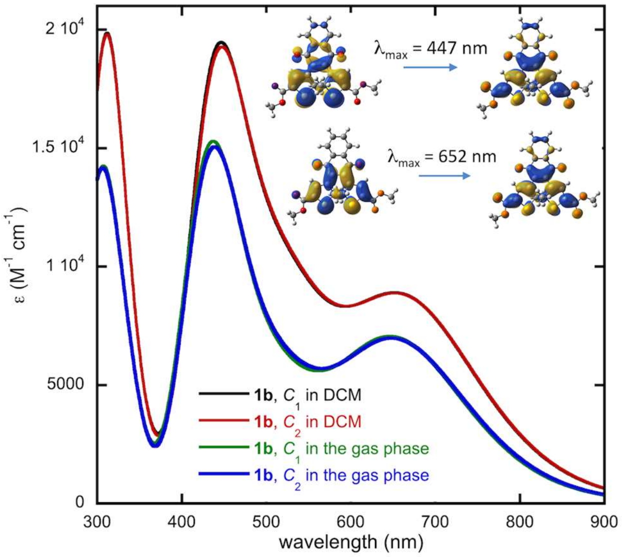

Figure S2. Computed UV-Vis spectra of the conformers for closed form isomer 1b in dichloromethane and in the gas phase at TD-B3LYP/6-31G* level and molecular orbitals involved in the main electronic transitions.

Figure S3 shows the computed spectra for the C_1 conformers of **1a** and **1b** along with the molecular orbitals involved in the main electronic transitions using CAM-B3LYP, which outperforms B3LYP for the computed TD-DFT spectra as expected. The spectrum of **1a** has the correct line shape with the first maximum at 387 nm, slightly blue-shifted compared to the experimental spectrum recorded for the methyl ester. This is in comparison to B3LYP, which gives the wrong line shape with two maxima, the longer wavelength maximum being a charge transfer state too low in energy (Figure S2). The CAM-B3LYP spectrum of **1b** presents two maxima at 571 and 407 nm. This is also consistent with the line shape of the experimental spectrum. Again, the absorption maxima are slightly blue-shifted in the calculated spectra.

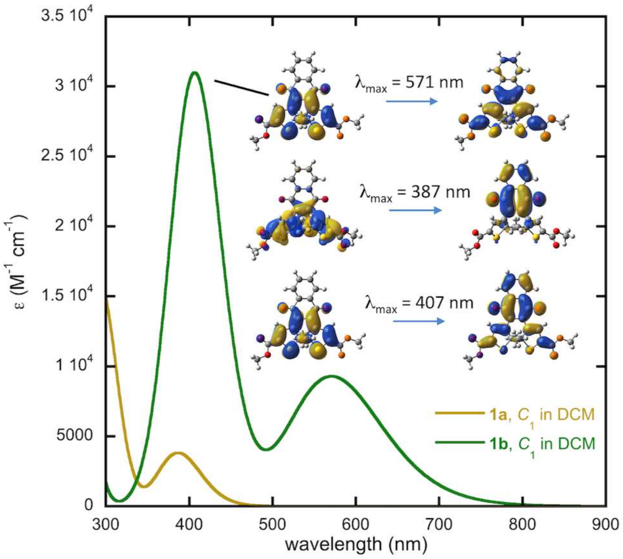

Figure S3. Computed spectrum of the C_1 conformers of 1a and 1b in DCM at TD-CAM-B3LYP/6-31G* level and molecular orbitals involved in the main electronic transitions.

To better approximate the line shapes observed, the basis set was changed from 6-31G* to 6-311+G**. A comparison of these two basis sets in presented in Figure S4 with the molecular orbital surfaces for the respective transitions shown. In the case of **1**, bathochromic shifts on moving to 6-311+G** are observed for both the ring open and ring closed forms much better matching the experimental data. For example, for **1a** the long wavelength absorption is now predicted to be 401 nm, close to the experimental value of 406 nm; for **1b**, the predicted long wavelength absorption is centered at 597 nm, closer to the actual value of 612 nm.

Given the results thus described, we arrived at the use of TD-DFT along with the CAM-B3LYP functional and the 6-311+G** basis set for calculations of the UV-vis absorption spectra of the naphthoquinone diarylethenes presented in our study.

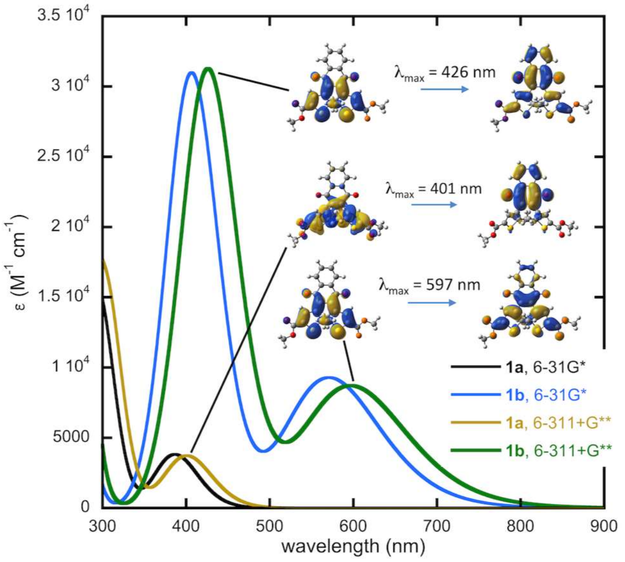

Figure S4. A comparison of TD-CAM-B3LYP/6-31G* and TD-CAM-B3LYP/6-311+G simulated spectra for the C_1 conformers of 1a and 1b. The respective transitions with the 6-311+G** basis set are shown.**

The t-butyl ester derivative **2** is expected to have a very similar thermal stability as methyl ester derivative **1**. Indeed, the energy difference between the open and closed forms of **2** is calculated at 42 kJ◎mol^{-1} and 48 kJ◎mol^{-1} in the gas phase and in DCM, respectively. The cyclization involves an activation energy of 173 kJ◎mol^{-1} in DCM, whereas that for the cycloreversion reaction is calculated at 126 kJ◎mol^{-1}. These results are very similar to those of compound **1** within a few kJ◎mol^{-1}.

Figure S5 shows the computed UV-vis absorption spectra for **2a** and **2b**. The spectra of **1a** and **1b** shown in Figure 7 are reported in this figure for a direct comparison. It is evident that the substitution of the methyl ester groups by t-butyl ester does not change significantly the spectra, as the position and intensity of the bands are barely changed and the nature of the main electronic transitions are unchanged. In conclusion, we expect the t-butyl ester nqDAE **2** to present very similar photochromic properties as the methyl ester derivative **1** and this is what is observed experimentally [1].

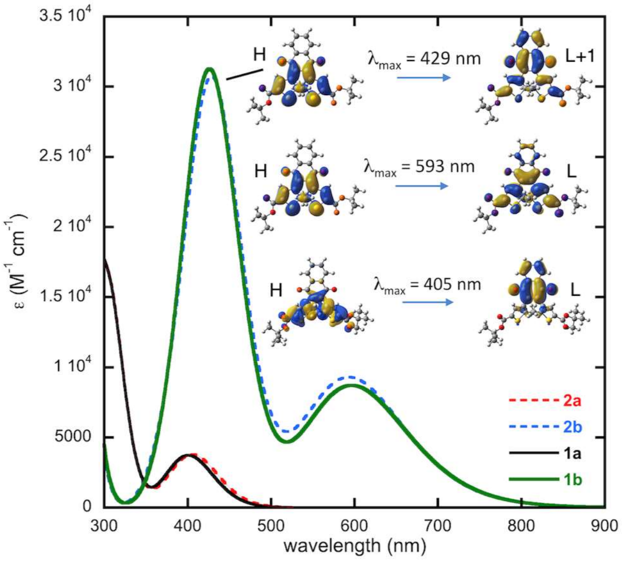

Figure S5. Computed spectrum of 2a and 2b in dichloromethane at TD-DFT level and molecular orbitals (H: HOMO, L: LUMO, isocontour: 0.02) involved in the main electronic transitions. The computed spectra of 1a and 1b are shown for comparison.

Crystallography Data

Table S1. Crystal data and structure refinement for Compound_5 and Compound_1b

Identification code	Compound_5	Compound_1b
Empirical formula	$C_{22}H_{14}O_2$	$C_{24}H_{18}O_6S_2$
Formula weight	310.33	466.50
Temperature/K	90	90
Crystal system	triclinic	monoclinic
Space group	P-1	$P2_1/c$
a/Å	11.1148(6)	7.1500(3)
b/Å	11.2447(6)	25.5032(9)
c/Å	15.3720(8)	22.9477(8)
α/°	70.3570(10)	90
β/°	70.4760(10)	93.5710(10)
γ/°	64.5080(10)	90
Volume/Å^3	1592.17(15)	4176.3(3)
Z	4	8
ρ_{calc}g/cm^3	1.295	1.484
μ/mm^{-1}	0.082	0.296
F(000)	648.0	1936.0
Crystal size/mm^3	0.1 × 0.08 × 0.06	0.2 × 0.05 × 0.04
Radiation	MoKα (λ = 0.71073)	MoKα (λ = 0.71073)
2Θ range for data collection/°	2.886 to 56.562	3.194 to 61.108
Index ranges	$-14 \le h \le 14$<br>$-14 \le k \le 14$<br>$-20 \le l \le 20$	$-10 \le h \le 10$<br>$-21 \le k \le 36$<br>$-32 \le l \le 19$
Reflections collected	31951	27949
Independent reflections	7875 [R_{int} = 0.0374, R_{sigma} = 0.0294]	12747 [R_{int} = 0.0363, R_{sigma} = 0.0607]
Data/restraints/parameters	7875/0/433	12747/0/663
Goodness-of-fit on F^2	1.025	1.047
Final R indexes [I>=2σ (I)]	R_1 = 0.0395, wR_2 = 0.0991	R_1 = 0.0506, wR_2 = 0.1155
Final R indexes [all data]	R_1 = 0.0474, wR_2 = 0.1050	R_1 = 0.0790, wR_2 = 0.1272
Largest diff. peak/hole / e Å^{-3}	0.37/-0.23	0.99/-0.46

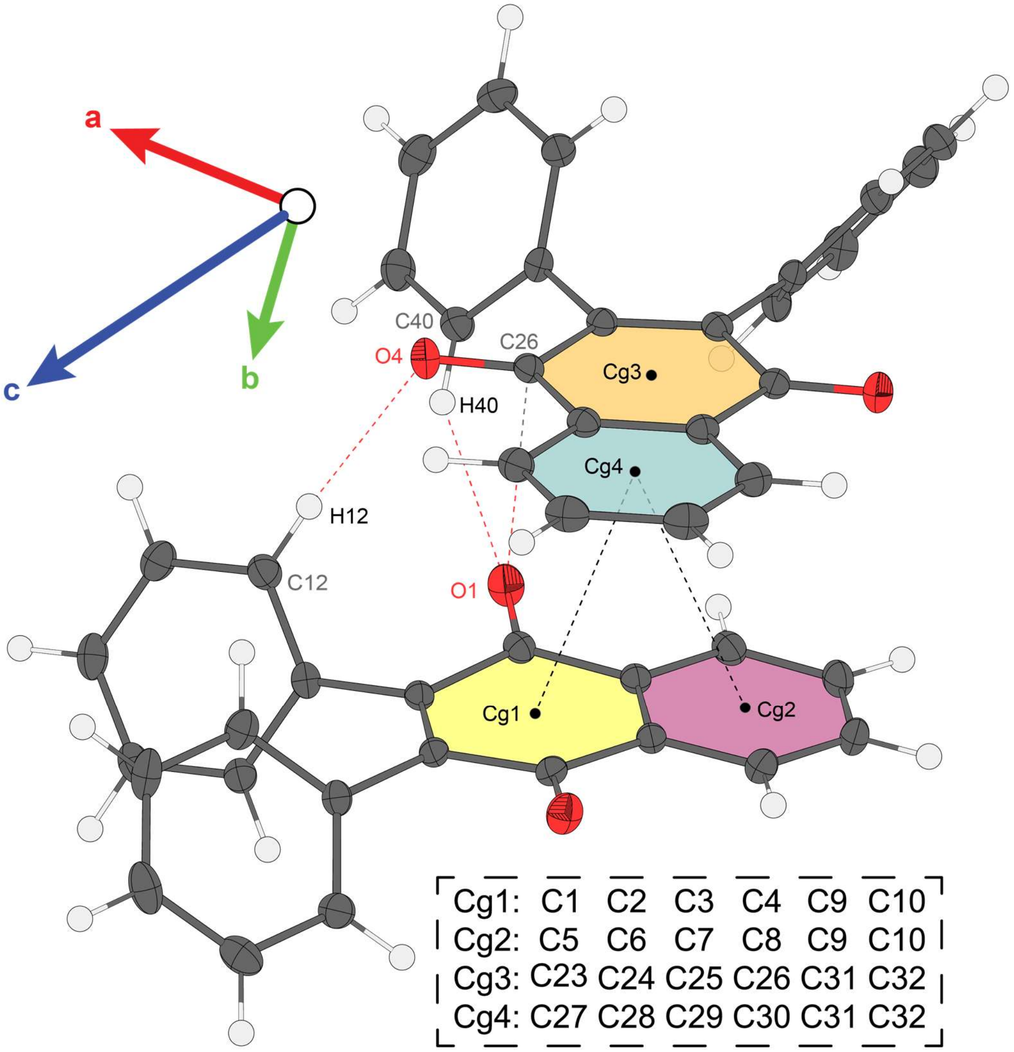

Figure S6. **ORTEP of phenyl nqDAE 5 illustrating the dimer formation through π-π stacking, hydrogen bonding, and electrostatic interactions. Atoms involved in hydrogen bonding and electrostatic interactions are labeled for clarity. Cg1, Cg2, Cg3, and Cg4 represent the center for each mean plane and their respective π systems.**

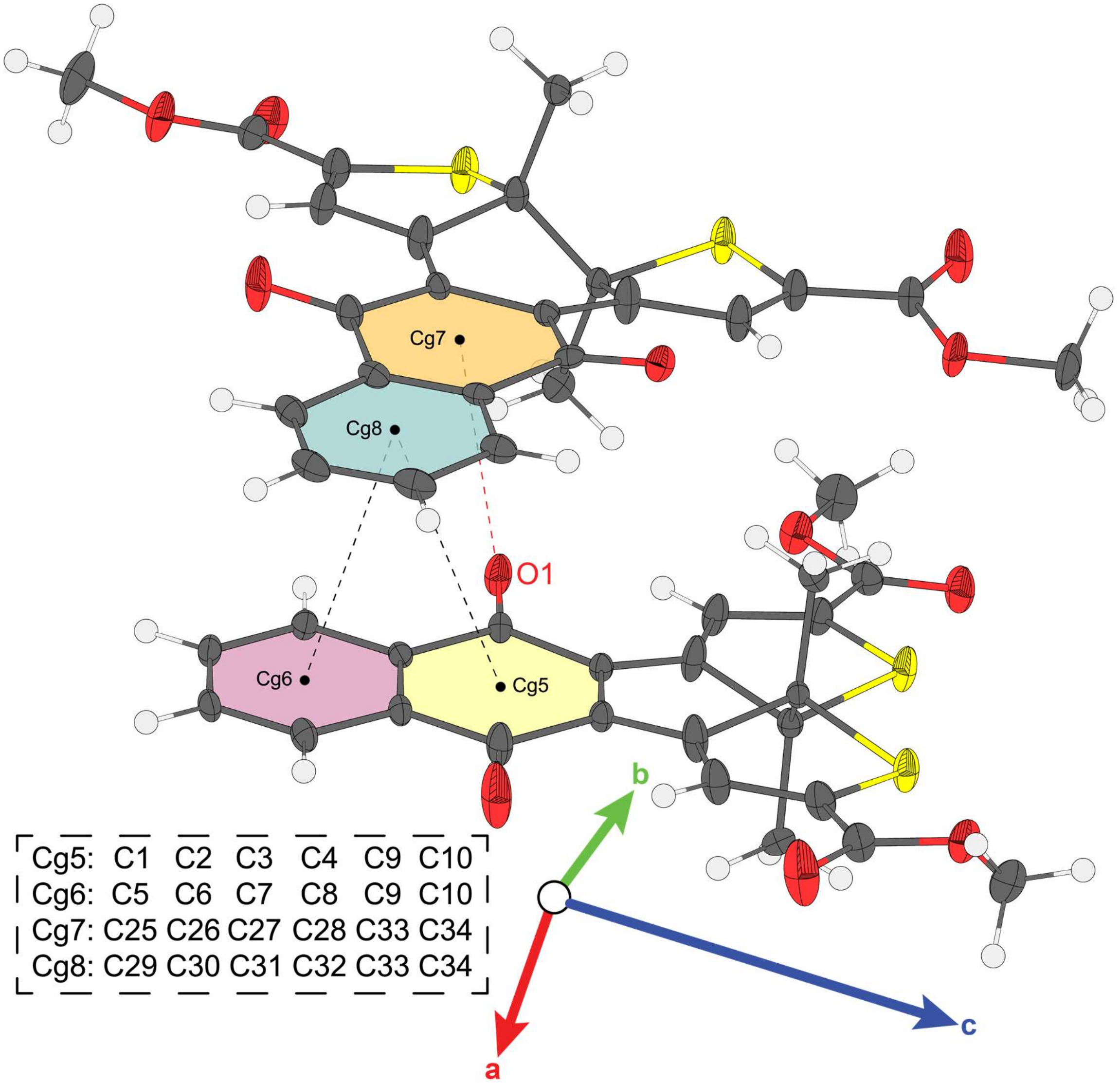

Figure S7. Interactions of the naphthoquinone backbone facilitating the stacking observed in the crystal structure of 1b with centroid-to-centroid distance and oxygen to centroid distances illustrated.

Table S2. π-πinteractions in the lattice of Compound_5 and Compound_1b[a]

Compound	Cg(I)	Cg(J)	Cg(I)···Cg(J) (Å)	α (°)[b]	β (°)[c]	Cg(I)_Perp (Å)[d]	Slippage (Å)[e]
**5**	Cg1	Cg4	3.6248(7)	4.23(5)	19.5	3.4377(5)	1.21
**5**	Cg2	Cg4	3.8068(7)	3.75(6)	26	3.4553(5)	1.67
**1b**	Cg5	Cg8	3.708(1)	3.06(9)	25.7	3.4003(8)	1.61
**1b**	Cg6	Cg8	3.552(1)	1.5(1)	19.9	3.3090(8)	1.21

a: Analysis of π-π interactions was performed using the CALC GEOM routine in PLATON [2] b: Dihedral angle betweenplanes I and J; c: Angle between Cg(I)-Cg(J) vector and normal to plane I; d: Perpendicular distance of Cg(I) on ring J.; e: Distance between Cg(I) and perpendicular projection of Cg(J) on ring I.

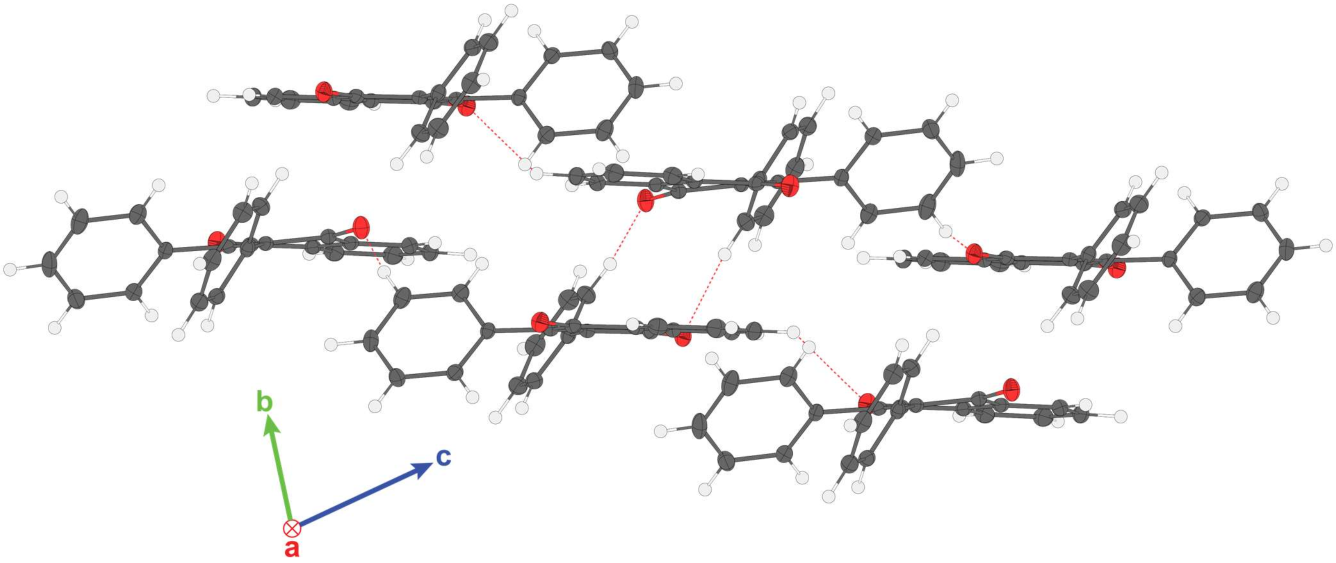

Figure S8. Extensive hydrogen-bonding network of 5 viewed down the crystallographic a-axis.

Table S3. Hydrogen Bond Geometry for 5 (Å, °)

D—H···A	D—H	H···A	D···A	D—H—A
C7—H7···O3^{i}	0.95	2.44	3.219(1)	139
C12—H12···O4	0.95	2.37	3.308(1)	169.3
C21—H21···O4^{ii}	0.95	2.35	3.209(2)	150.4
C28—H28···O2^{iii}	0.95	2.49	3.261(2)	138.1
C37—H37···O1^{iv}	0.95	2.34	3.243(1)	158
C40—H40···O1	0.95	2.47	3.321(2)	149.2

Symmetry Codes: (i) -x, 1-y, 1-z; (ii) 1-x, -y, 2-z; (iii) -x, -y, 2-z; (iv) 1-x, 1-y, 1-z

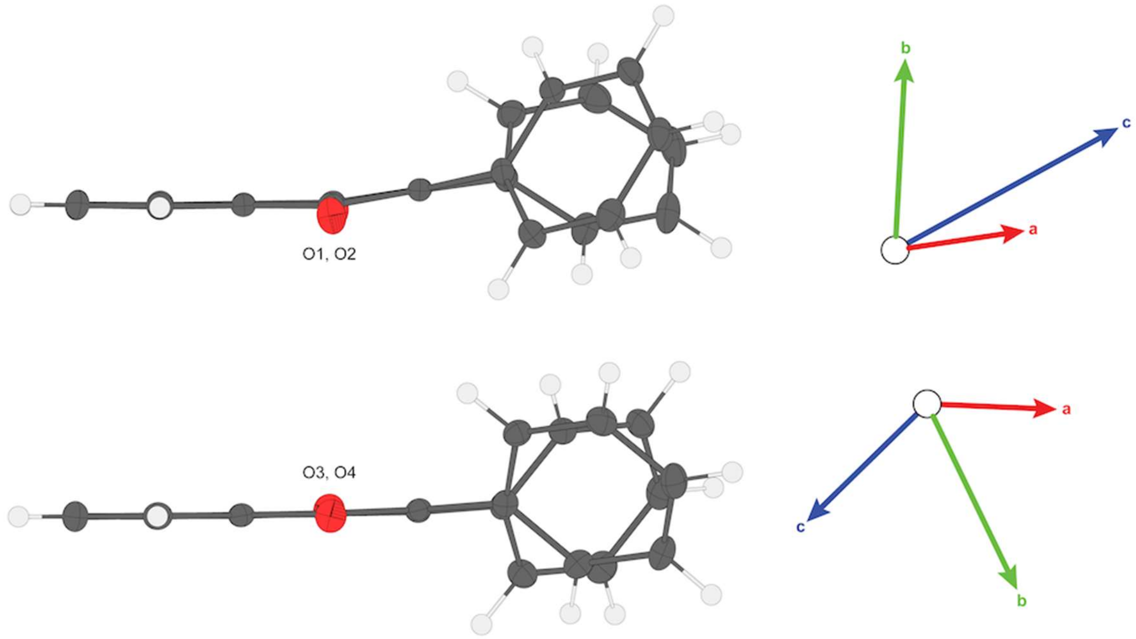

Figure S9. Comparison of the non-planar (top) and planar (bottom) molecules present in the crystal structure of compound 5. Planarity refers to the naphthoquinone portion of the molecules.

Cartesian Coordinates for Computations

Table S4. Optimized B3LYP/6-31G* Cartesian coordinates (in Å) for C_1 conformer of compound 1a in dichloromethane.

E = -2173.144563 a.u.

S	2.601059029328	-0.179084286543	-2.560303002301
S	-2.772490451931	-1.540802079289	-2.033748937158
O	-3.559252342641	-4.369562657098	-2.184491057932
O	2.141477594425	2.077830200334	1.648179690260
C	0.536659871776	0.535857092112	0.824422041199
O	3.358676631627	2.162284953292	-4.355336710226
O	2.393234486200	3.741900979143	-3.045197943880
C	0.576338787953	1.352451971662	3.264656352744
C	1.161024475829	1.383151734196	1.900189620296
C	-0.597473473605	-0.183078806693	1.073203966179
C	-0.546069618210	0.553909059969	3.538392964118
C	-1.079113202418	0.519479746202	4.831464382913
H	-1.947209292689	-0.101728255423	5.023551752864
C	1.555168320098	1.740235659902	-1.192366560310
H	1.253129269469	2.727764651807	-0.868808013902
C	2.256531637280	1.518729591400	-2.349634742912
C	0.621013831051	2.075900055680	5.571578870470
H	1.073386564660	2.666136351914	6.362892655519
O	-2.589018937992	-5.096207313165	-0.267394798959
C	-0.496227034171	1.278432771611	5.844809255106
H	-0.911462835841	1.250818188857	6.847809159283
C	-1.181665020255	-0.243038540691	2.458780821224
O	-2.171399825343	-0.934131669311	2.682340871829
C	-1.661066743869	-2.323237484260	0.155241598351
H	-1.337663573283	-2.950663447789	0.976078063019
C	1.156450178211	2.113564207308	4.285295707764
H	2.023692281137	2.723178150435	4.055787307086
C	2.731369602544	2.474148480306	-3.355158782694
C	-2.399380661925	-2.809424524984	-0.892406727814
C	-4.037928040082	-5.705083269889	-2.430364310360
H	-4.590234731630	-5.647518671874	-3.367732278232
H	-4.692860188709	-6.030557494012	-1.618561551188
H	-3.199382072366	-6.399759744523	-2.521574088365
C	1.254127232933	0.541944856418	-0.472233797718
C	-1.354538600082	-0.931761974866	0.042254409499
C	-2.839921484376	-4.199566488802	-1.054446762283
C	2.829659931894	4.748965378348	-3.977802824600
H	3.919792089817	4.753175921042	-4.052872551920
H	2.471175535260	5.694219893090	-3.571353810815

H	2.398562684604	4.568100474041	-4.965475763041
C	-1.907588404820	-0.360764682119	-1.094384462916
C	1.774474132095	-0.587516200842	-1.087212179862
C	1.736858947674	-2.015622471831	-0.632651863678
H	2.669278623077	-2.533595112021	-0.877190008729
H	0.915099992061	-2.565286397416	-1.106532171712
H	1.590314029565	-2.069290010877	0.449533569771
C	-1.874109096542	1.067117617016	-1.550369033920
H	-2.818039627411	1.354358427340	-2.023822118327
H	-1.070632121073	1.236565361622	-2.276860976567
H	-1.700618579307	1.735673309269	-0.702620137172

Table S5. Optimized B3LYP/6-31G* Cartesian coordinates (in Å) for C_2 conformer of compound 1a in dichloromethane.

E = -2173.144397 a.u.

S	2.206462519810	1.706452035264	2.274968384316
S	-2.206462519602	-1.706452035264	2.274968384516
O	2.572034034127	0.754739014448	-2.409276072458
O	5.168665272956	-0.813072311448	1.547598175279
O	4.892084617805	0.654580455301	3.254542125527
C	1.392792664758	0.413272070074	-2.422132238154
O	-5.168665272815	0.813072311448	1.547598175749
C	0.645792274998	0.222126943066	-1.129629438793
O	-2.572034034346	-0.754739014448	-2.409276072223
C	-0.645792275101	-0.222126943066	-1.129629438734
C	1.346925214222	0.380711679083	-4.915964019624
H	2.389652354489	0.678481751100	-4.896489006939
O	-4.892084617509	-0.654580455301	3.254542125972
C	-0.673501252132	-0.189997424163	-6.121267746421
H	-1.195965749665	-0.338127654797	-7.061634044240
C	-3.230353996901	-0.485814335916	1.562784959251
C	-0.675506095135	-0.191719721200	-3.703145688226
C	-2.681546245753	0.005502614697	0.406259699875
H	-3.164038323550	0.763405743160	-0.196972423393
C	4.494525608381	0.141013597081	2.220473199309
C	0.673501251575	0.189997424163	-6.121267746482
H	1.195965749022	0.338127654797	-7.061634044349
C	-1.392792664978	-0.413272070074	-2.422132238027
C	3.230353997044	0.485814335916	1.562784958957
C	0.675506094797	0.191719721200	-3.703145688288
C	-1.410584263296	-0.569598444826	0.091235594070
C	-1.346925214669	-0.380711679083	-4.915964019501
H	-2.389652354934	-0.678481751100	-4.896489006722
C	-1.025306821634	-1.533077858728	1.012131338781
C	2.681546245790	-0.005502614697	0.406259699631
H	3.164038323532	-0.763405743160	-0.196972423681
C	-4.494525608179	-0.141013597081	2.220473199718
C	1.410584263304	0.569598444826	0.091235593942
C	6.429406518243	-1.207876168460	2.121244394363
H	6.282824900269	-1.610863509602	3.126307839306
H	7.111362129708	-0.355367351200	2.168002007833
H	6.822505577024	-1.975275037737	1.455094486600
C	0.194301328387	-2.404902597686	1.016860853807
H	-0.034464357717	-3.404066794507	1.399966170534

H	0.987179652706	-1.980051095667	1.643643724231
H	0.593443189034	-2.510823421598	0.004395498533
C	1.025306821726	1.533077858728	1.012131338688
C	-6.429406518050	1.207876168460	2.121244394948
H	-6.822505576891	1.975275037737	1.455094487221
H	-6.282824899985	1.610863509602	3.126307839878
H	-7.111362129511	0.355367351200	2.168002008481
C	-0.194301328295	2.404902597686	1.016860853825
H	0.034464357844	3.404066794507	1.399966170531
H	-0.987179652556	1.980051095667	1.643643724321
H	-0.593443189033	2.510823421598	0.004395498587

Table S6. Optimized B3LYP/6-31G* Cartesian coordinates (in Å) for C_1 conformer of compound 1b in dichloromethane.

E = -2173.125687 a.u.

S	0.153692379836	1.011840000103	-3.030056451777
S	-0.123507158126	-2.458812093887	-2.102551364416
O	0.016068345519	-5.362489264137	-2.047779603590
O	-0.240464420541	3.238498291755	1.778773956039
C	0.028911446351	0.992793829939	1.005077535552
O	-0.006407415283	3.665331149744	-4.419230418171
O	0.052459729733	4.937767749954	-2.543834071075
C	-0.055639284694	1.612185838555	3.473217623496
C	-0.107808474172	2.041928873438	2.049364994162
C	0.008540616886	-0.430863885607	1.384628101734
C	0.086536500405	0.259630852654	3.833634246136
C	0.154462434571	-0.093102826550	5.189116573805
H	0.262117333290	-1.141304906801	5.445063790390
C	0.148777370631	2.635452746980	-0.931591260905
H	0.095462503482	3.549471418316	-0.361082908842
C	0.124883028368	2.606275473042	-2.293839742111
C	-0.064174216040	2.232307789903	5.815944545208
H	-0.122317986815	2.996764799020	6.585232782512
O	-0.026007659847	-5.702056441822	0.193174418597
C	0.080047620963	0.885387933043	6.174741165496
H	0.132148368977	0.604325368918	7.222532125414
C	0.147137150170	-0.821009739986	2.812111662468
O	0.287040366331	-1.992584441784	3.172392338744
C	-0.116233574860	-2.819078175024	0.523440105777
H	-0.063897117377	-3.335088712957	1.469713405563
C	-0.130712033069	2.591672268981	4.473992477828
H	-0.237916178583	3.628446407132	4.175048911669
C	0.051991023972	3.772373216689	-3.206745187438
C	-0.096081821718	-3.476637733140	-0.669882124808
C	0.082398965161	-6.788294364745	-2.257939113498
H	0.117734745519	-6.919757717659	-3.338361399470
H	-0.802475697274	-7.272292045056	-1.838507006262
H	0.980557306131	-7.196692525843	-1.789168074601
C	0.186619931383	1.339529355809	-0.322190580017
C	-0.151219261680	-1.392637066258	0.406734959631
C	-0.034471149732	-4.957122458497	-0.768673373130
C	-0.029030750397	6.124193851173	-3.361337544287
H	0.829001379114	6.178457170231	-4.035237898109
H	-0.021244902680	6.957538006987	-2.660271946451

H	-0.952734063921	6.118531162582	-3.944494417555
C	-0.443520510943	-0.950859065898	-1.033345746230
C	0.476517876027	0.239453387520	-1.352463520001
C	1.991118219915	-0.095270104970	-1.297280286002
H	2.256572796181	-0.833785823618	-2.056316640433
H	2.264414518843	-0.488130180306	-0.313970727421
H	2.569188744789	0.814513379573	-1.477139605842
C	-1.958265511253	-0.633240887394	-1.150066657005
H	-2.225422880646	-0.370841094999	-2.175610155211
H	-2.230254406689	0.197462892277	-0.492861347990
H	-2.535893756207	-1.511470457384	-0.851253377571

Table S7. Optimized B3LYP/6-31G* Cartesian coordinates (in Å) for C_2 conformer of compound 1b in dichloromethane.

E = -2173.125578 a.u.

S	1.796314105053	0.125158401148	2.742497984990
S	-1.796314105053	-0.125158401148	2.742497984990
O	2.705400770619	-0.278981129783	-2.476857677984
O	5.463070656221	0.008428493522	1.259382964354
O	4.718835925466	-0.044645751018	3.400211139255
C	1.480604495952	-0.136403555854	-2.430098584564
O	-5.463070656221	-0.008428493522	1.259382964354
C	0.736776523843	0.005175922118	-1.151116341145
O	-2.705400770619	0.278981129783	-2.476857677984
C	-0.736776523843	-0.005175922118	-1.151116341145
C	1.388118548546	-0.152021080208	-4.914544506741
H	2.466110708904	-0.266796227737	-4.892563654920
O	-4.718835925466	0.044645751018	3.400211139255
C	-0.696416680019	0.076939974944	-6.118821369106
H	-1.236999638435	0.135956402528	-7.058954676695
C	-3.146639061846	-0.089001235669	1.620314554386
C	-0.699408747476	0.075721408562	-3.695372495653
C	-2.823838775323	-0.113477826638	0.296472536801
H	-3.559640541526	-0.056184095402	-0.490329464509
C	4.508810673904	0.012090602450	2.201223725417
C	0.696416680019	-0.076939974944	-6.118821369106
H	1.236999638435	-0.135956402528	-7.058954676695
C	-1.480604495952	0.136403555854	-2.430098584564
C	3.146639061846	0.089001235669	1.620314554386
C	0.699408747476	-0.075721408562	-3.695372495653
C	-1.414857559453	-0.158900618306	0.041940974283
C	-1.388118548546	0.152021080208	-4.914544506741
H	-2.466110708904	0.266796227737	-4.892563654920
C	-0.619769510534	-0.455238624732	1.320625269009
C	2.823838775323	0.113477826638	0.296472536801
H	3.559640541526	0.056184095402	-0.490329464509
C	-4.508810673904	-0.012090602450	2.201223725417
C	1.414857559453	0.158900618306	0.041940974283
C	6.820385969623	-0.074669009315	1.742242790770
H	6.964887027534	-0.998858086275	2.306471526323
H	7.047788011859	0.782765259016	2.379720365608
H	7.444201196042	-0.066995115504	0.849604790949
C	-0.293675534185	-1.972500653515	1.352433818481
H	0.222245343742	-2.242611482912	2.275894837084

H	0.337218791860	-2.249925109252	0.503324511871
H	-1.223406020591	-2.543519794356	1.291246441806
C	0.619769510534	0.455238624732	1.320625269009
C	-6.820385969623	0.074669009315	1.742242790770
H	-7.444201196042	0.066995115504	0.849604790949
H	-6.964887027534	0.998858086275	2.306471526323
H	-7.047788011859	-0.782765259016	2.379720365608
C	0.293675534185	1.972500653515	1.352433818481
H	-0.222245343742	2.242611482912	2.275894837084
H	-0.337218791860	2.249925109252	0.503324511871
H	1.223406020591	2.543519794356	1.291246441806

Table S8. Optimized broken-symmetry B3LYP/6-31G* Cartesian coordinates (in Å) for transition state between 1a and 1b in dichloromethane.

S	0.963677997240	2.962074824828	-0.750123245400
S	-2.461946171465	2.000812534731	0.699164702939
O	3.073420474053	-1.780198697367	-0.952057862428
C	1.967662970445	-2.089249626054	-0.505001711431
C	0.928848746431	-1.053729237052	-0.246523647899
O	-1.778897327838	-3.138967303634	0.953134245004
C	-0.331242933933	-1.407070460093	0.226936659798
C	2.587871197222	-4.502995460511	-0.468607680441
H	3.565794447424	-4.208129010862	-0.833399895414
C	1.009847965537	-6.197263080986	0.235947894162
H	0.770700992707	-7.240621486954	0.419097778533
C	-3.445228030587	0.644459493343	0.200960200446
C	0.353140974803	-3.868409728542	0.233518885679
C	-2.715527630324	-0.535694867240	0.143800575223
H	-3.167874741251	-1.467957094750	-0.164719105286
C	2.279724076912	-5.841456783379	-0.232385642218
H	3.027037148989	-6.608450545828	-0.412909084034
C	-0.676474794237	-2.830114869395	0.498143045753
C	2.508755134291	2.323182465464	-0.246015894718
C	1.628339705046	-3.511035134365	-0.237368453198
C	-1.356921571565	-0.388697292683	0.451677113085
C	0.049952825080	-5.214047459174	0.468618802795
H	-0.938906385540	-5.470015509849	0.833215867402
C	-0.982237058214	0.987138346704	0.774953524637
C	2.506871652295	0.936184137404	-0.175766280523
H	3.375345734992	0.374030161001	0.137003846052
C	1.271250929429	0.348903435399	-0.481421310337
C	-0.125258265965	1.294283757925	1.993904859842
H	0.770960226626	0.672243318545	2.014066830698
H	-0.699959825335	1.088012569943	2.902425291092
H	0.175912467040	2.345610839100	2.013237562219
C	0.233894287965	1.324235911930	-0.814123380645
C	-0.655837662621	1.128750046703	-2.032707118818
H	-0.055962312958	1.242427220927	-2.941253987876
H	-1.459153528602	1.870589355305	-2.060796653387
H	-1.098584606430	0.131599334411	-2.043997999729
C	3.588385305573	3.255133591930	0.081954639858
O	3.477598360000	4.471783681526	0.033603765848
O	4.720648690765	2.613486851795	0.436220414344

E = -2173.075435 a.u.
<S^2> = 0.877207
ν^{TV} = 739*i* cm^{-1}

E(triplet) = -2173.070847 a.u.
<S^2> = 2.028322

C	5.840660366017	3.458800716111	0.760653072316
H	5.600551718677	4.100899625523	1.611680905465
H	6.653286334985	2.778102026027	1.012296613961
H	6.111653795899	4.079202022289	-0.097112292678
C	-4.861059590755	0.763877985724	-0.153695312110
O	-5.565626557129	-0.175792139637	-0.487687286648
O	-5.296291064097	2.038712371870	-0.059386923078
C	-6.682185455448	2.255097770169	-0.384884151771
H	-7.324881896369	1.683017793806	0.288653312966
H	-6.847234913306	3.324141143618	-0.255363566231
H	-6.881881767249	1.958201299279	-1.417372437103

Table S9. Optimized B3LYP/6-31G* Cartesian coordinates (in Å) for C_1 conformer of compound 2a in dichloromethane.

E = -2409.045145 a.u.

S	2.128896832711	1.784554144240	-1.913220398983
S	-1.355894757420	-2.535492565259	-1.366518583477
O	-0.350643750187	-5.243968427785	-1.821065801617
O	0.816875152199	3.017631838595	2.535638209325
C	0.362930739463	0.887076642012	1.593808338061
O	1.212727898339	4.206020039715	-3.465839917446
O	-0.398246706554	4.814793018456	-1.959250090484
C	0.135796662093	1.389066271165	4.107752598484
C	0.463071490392	1.859381503658	2.737715699540
C	-0.098874452102	-0.382425750808	1.796178803828
C	-0.264033486021	0.060622191613	4.325860561438
C	-0.553302372633	-0.376711328768	5.622944437647
H	-0.862305030038	-1.405659089652	5.771835679927
C	0.282808532253	2.599790002754	-0.309499041399
H	-0.513136625840	3.186374346467	0.130182589600
C	0.869058444178	2.922397511323	-1.505863757097
C	-0.049399998863	1.831394497973	6.477907860830
H	0.033870416932	2.517825848830	7.315240855789
O	1.063153292304	-5.385263895058	-0.027226335997
C	-0.445828917578	0.506890702500	6.695507789655
H	-0.671431812945	0.165831788013	7.701504151038
C	-0.397317295835	-0.880022014338	3.184460924612
O	-0.752439511449	-2.041836285781	3.361614972376
C	0.215993896018	-2.661273176663	0.670133099545
H	0.926548109144	-3.031282923190	1.398202180163
C	0.240826265810	2.272097811016	5.187872219139
H	0.552776316863	3.293811203976	5.000332112494
C	0.594984865472	4.039477543336	-2.423353133107
C	-0.196327189867	-3.413302737575	-0.398868053316
C	-0.083081045687	-6.595892313408	-2.364716324159
C	0.815014195351	1.411752179946	0.283741833262
C	-0.366565164351	-1.356269282724	0.711788085760
C	0.251045036013	-4.781178975710	-0.709944342581
C	-0.871744611828	6.014271799144	-2.689572833576
C	-1.259986193126	-1.144360757023	-0.327597193259
C	1.842574354639	0.861757692815	-0.468391824166
C	2.696199979180	-0.335071296149	-0.173638767729
H	3.727329090296	-0.177601421542	-0.504738309539
H	2.316736295697	-1.231681556427	-0.677759691862

H	2.710294374844	-0.540758769582	0.900244937351
C	-2.116851008974	0.052903181065	-0.611402653457
H	-3.089365739717	-0.242732859234	-1.016930057310
H	-1.641205695535	0.722673417398	-1.337573500918
H	-2.289249705710	0.626084774495	0.303877608138
C	-0.498398828340	-7.657211657830	-1.341769817496
H	-0.415190237385	-8.650335486303	-1.796501883859
H	0.136378088159	-7.625179585863	-0.454849657238
H	-1.540358853943	-7.508077952070	-1.038853011495
C	1.390596631201	-6.708755814018	-2.765661189555
H	1.554758224097	-7.663458031576	-3.277113603585
H	1.661032096392	-5.902416385241	-3.455900830407
H	2.044786667737	-6.663071334164	-1.893475449847
C	-0.988455509375	-6.642947651856	-3.597161971093
H	-0.882451011443	-7.609522208197	-4.099954305129
H	-2.037703457007	-6.513862921739	-3.312605877730
H	-0.720122354051	-5.852714170713	-4.305745420725
C	-1.978848758383	6.541156856479	-1.774400632080
H	-1.578789813602	6.790842003346	-0.786400276506
H	-2.421310277566	7.444024717621	-2.207299304060
H	-2.768888954142	5.793225711023	-1.651382228101
C	0.265917764691	7.032203012470	-2.810066944118
H	1.063965994524	6.662547053283	-3.455818262054
H	-0.125239983733	7.963309495898	-3.234441883296
H	0.682724672641	7.257178786618	-1.822481873196
C	-1.439507806163	5.601781082864	-4.050663706194
H	-2.211990279075	4.835293320386	-3.925707027098
H	-1.899290343975	6.472755135406	-4.530183847171
H	-0.658361609187	5.214166110346	-4.706523994492

Table S10. Optimized B3LYP/6-31G* Cartesian coordinates (in Å) for C_1 conformer of compound 2b in dichloromethane.

S	0.120364903359	1.152730789519	-2.463179064555	E = -2409.027157 a.u.
S	-0.169300735131	-2.353016831886	-1.691076989858	
O	-0.023311337393	-5.241543752801	-1.771716746574	
O	-0.248686460591	3.165254594256	2.441498112829	
C	0.010147535803	0.955357469924	1.567252139952	
O	-0.028035341502	3.832616701748	-3.742922192316	
O	0.040818487778	5.043965460652	-1.804750055314	
C	-0.065280433632	1.463586679078	4.060357654134	
C	-0.119496556288	1.956807054268	2.656975995629	
C	-0.011651897767	-0.484318605501	1.883139972108	
C	0.076095513862	0.096157727617	4.359591482194	
C	0.147029756703	-0.316649560820	5.697934924474	
H	0.254167795701	-1.375313661470	5.906830221059	
C	0.128807706228	2.681993829453	-0.296204329604	
H	0.081139048340	3.570752216547	0.313019941721	
C	0.100498645211	2.714303342555	-1.658261830379	
C	-0.067761634409	1.978601129445	6.428558254841	
H	-0.123455423042	2.708033284905	7.231356683449	
O	-0.047740794146	-5.681443991354	0.472000425182	
C	0.076062824608	0.616849540677	6.726484700414	
H	0.130418068061	0.289173525769	7.760562067512	
C	0.132153275927	-0.937967753646	3.290254747533	
O	0.271780980592	-2.124732668932	3.598306626752	
C	-0.141875037437	-2.831644228419	0.914672373910	
H	-0.082105775420	-3.390419892074	1.835806192241	
C	-0.137018424551	2.397435987992	5.104148227459	
H	-0.243792936030	3.446618712005	4.851883918536	
C	0.031470147443	3.921210771558	-2.526862895770	
C	-0.129603987809	-3.435183255289	-0.307051550609	
C	0.049931483879	-6.651931545776	-2.238165455319	
C	0.163799927443	1.360619581973	0.255890244649	
C	-0.177538989166	-1.401458285226	0.863792566870	
C	-0.064464893668	-4.915115996799	-0.475061624631	
C	-0.027659664807	6.392278357680	-2.430214516488	
C	-0.476714555519	-0.894296996365	-0.553073822858	
C	0.445766893069	0.306226426895	-0.822984146045	
C	1.959360379474	-0.036223913879	-0.788123460442	
H	2.218657971185	-0.743456514998	-1.578517475383	
H	2.235120858190	-0.471022216108	0.176669997048	

H	2.540265015235	0.878129079073	-0.932230436924
C	-1.990712277756	-0.565502890892	-0.645855986245
H	-2.262492164764	-0.253454278091	-1.656223019228
H	-2.255691071638	0.234063494547	0.051596012812
H	-2.570296190466	-1.454771028431	-0.385890670326
C	-1.201036712129	-7.406864814253	-1.781901314412
H	-1.200268962646	-8.406526244610	-2.229464288885
H	-1.230759712972	-7.512174006290	-0.696096954609
H	-2.104957154239	-6.885827069246	-2.114730938382
C	1.345210593262	-7.290831197066	-1.731178714204
H	1.340385276814	-7.396432298638	-0.645033642043
H	1.454141131628	-8.284854445855	-2.177914721379
H	2.210388141013	-6.688206120781	-2.027231734271
C	0.072986812427	-6.495288974081	-3.759116404362
H	0.126714181980	-7.481173962327	-4.231668692058
H	-0.832688480934	-5.991205797586	-4.111051997455
H	0.942945582233	-5.911326041175	-4.075991840208
C	0.007125290507	7.318657410826	-1.214060778667
H	-0.038563998111	8.361886493911	-1.542265701425
H	-0.844824443768	7.125259129095	-0.554488623646
H	0.929929972901	7.175130439902	-0.643035284327
C	-1.346078111469	6.538067390438	-3.194001817406
H	-1.446813263910	7.571677139462	-3.542120020825
H	-1.382829598338	5.874738787618	-4.059713850515
H	-2.194550678913	6.316171345391	-2.538110338977
C	1.199526379916	6.606503299094	-3.319660796347
H	1.209233738336	7.642319553800	-3.675505277403
H	2.119104446788	6.434112210835	-2.750399354729
H	1.187357714467	5.941862802155	-4.185120077906

Table S11. Optimized broken-symmetry B3LYP/6-31G* Cartesian coordinates (in Å) for transition state between 2a and 2b in dichloromethane.

S	1.081618274914	2.385278152055	-0.900021773156
S	-2.309764654025	1.595062396911	0.730863160930
O	2.984899596730	-2.444128844081	-1.096378086801
C	1.887788480222	-2.696968508194	-0.594988275218
C	0.905381651066	-1.614746365862	-0.312882144575
O	-1.830875782098	-3.564261573854	1.044721232124
C	-0.346573340714	-1.906486906673	0.223991640494
C	2.409357567626	-5.133688118475	-0.537507858410
H	3.381690846397	-4.886365131705	-0.949544666969
C	0.795071524457	-6.747668218026	0.266178236344
H	0.521214909971	-7.776795389854	0.479042269624
C	-3.371265536502	0.273760693122	0.304418889989
C	0.235586904989	-4.393546844974	0.247572295723
C	-2.694297218571	-0.935943679075	0.234854490428
H	-3.199133730354	-1.852895911334	-0.035466638189
C	2.056952079888	-6.453718437664	-0.262885265173
H	2.763332478721	-7.254490185078	-0.460820854927
C	-0.737861796873	-3.308294043953	0.536417927370
C	2.622148078683	1.691660032968	-0.455180732312
C	1.502500684254	-4.098347832005	-0.284488228880
C	-1.316831328726	-0.842036954453	0.476495619634
C	-0.112232220123	-5.721086502478	0.521217284328
H	-1.093912619558	-5.928875265705	0.932962182505
C	-0.871401321742	0.521815540257	0.756455646001
C	2.565845777109	0.308290271524	-0.357499168121
H	3.424501442543	-0.283431033690	-0.074178111431
C	1.293361865829	-0.233034957902	-0.592917245540
C	0.056568190718	0.814508501682	1.925650288998
H	0.928328132798	0.158416068652	1.912783940149
H	-0.479398468633	0.645870199228	2.865182783979
H	0.398402876831	1.853506265879	1.910923064254
C	0.283131212122	0.778882734324	-0.897345544479
C	-0.672128718244	0.598075450241	-2.067346224885
H	-0.113795411960	0.671914972758	-3.006063596253
H	-1.446305986622	1.370805682804	-2.070868902828
H	-1.153674514842	-0.380555933400	-2.038070296346
C	3.753697848911	2.591527978667	-0.195637294838
O	3.655335899256	3.809844853216	-0.270090430702
O	4.863160260485	1.900943328613	0.115164082114

E = -2408.976231 a.u.
$\langle S^2 \rangle$ = 0.887184
ν^{TV} = 739*i* cm^{-1}

E(triplet) = -2408.971925 a.u.
$\langle S^2 \rangle$ = 2.02789

C	6.155110171696	2.569187078112	0.401443928140
C	-4.801588045032	0.447848055911	0.014447468975
O	-5.537070921666	-0.486350217785	-0.270562591653
O	-5.159760507023	1.740927821584	0.115602348000
C	-6.548533107212	2.200449054794	-0.121933947596
C	7.083225409139	1.385606202864	0.681344055779
H	7.143681770888	0.725981309247	-0.190264316795
H	8.089977688702	1.748649204753	0.911575603841
H	6.721826465694	0.802726923154	1.534742937461
C	6.620139664024	3.347465057171	-0.832674508020
H	5.958285826999	4.188361767419	-1.046192785760
H	7.630498711291	3.732835122192	-0.657685455724
H	6.653827329819	2.689823501386	-1.707899941105
C	6.014377323124	3.457924495338	1.640617812613
H	5.344063885365	4.298400015922	1.453624348199
H	5.629369265080	2.876666577033	2.485412931343
H	6.998709293937	3.850270013447	1.918496729350
C	-7.487359029625	1.556751878079	0.902447423625
H	-8.487262236378	1.992341106091	0.800386095142
H	-7.559862379633	0.478146764002	0.753511178255
H	-7.133342593381	1.752384511331	1.920311198579
C	-6.954140150867	1.894963583157	-1.566655209653
H	-7.022545392366	0.820165288623	-1.742573136594
H	-7.931395635288	2.345822094835	-1.770620673406
H	-6.228802107204	2.324810602463	-2.265818637361
C	-6.442248538032	3.709304803483	0.107310858211
H	-7.420570913973	4.177518261511	-0.040764217553
H	-6.106152065329	3.923844835795	1.126880894442
H	-5.733063057683	4.159171213620	-0.594968315688

Table S12. Optimized B3LYP/6-31G* Cartesian coordinates (in Å) for C_1 conformer of compound 3a in dichloromethane.

E = -2094.532849 a.u.

S	1.426453848754	1.529042422716	-3.086425776540
S	-1.416524562770	-3.037909913831	-1.624816840649
O	-0.113198196684	-5.680285083233	-1.846961968209
O	0.422309176541	3.195619860671	1.299112881302
C	0.142631806784	0.920980424797	0.684085067007
O	0.033733744125	3.643395511104	-4.794913367252
O	-1.450217268789	4.254643057738	-3.201080608316
C	0.109074755349	1.729341773171	3.127059322496
C	0.236201365598	2.044059726029	1.681820534888
C	-0.138431556242	-0.351641828714	1.091237037319
C	-0.102441723334	0.406824149527	3.549980548977
C	-0.203091502471	0.118755543248	4.915344632442
H	-0.367792299353	-0.908134729550	5.223140671837
C	-0.334900984218	2.345745738183	-1.388513214607
H	-1.148336175302	2.895539870917	-0.933150918299
C	0.086339581486	2.567516616177	-2.675057709956
C	0.113115692943	2.462859926162	5.431179981786
H	0.196955002784	3.260279493453	6.163484647124
O	1.443045360205	-5.417421248812	-0.226653940254
C	-0.095133433511	1.144623894359	5.852698775083
H	-0.173958710972	0.919311272351	6.912035302912
C	-0.235425281277	-0.687230537980	2.555234047115
O	-0.428967753039	-1.846051484813	2.911193589218
C	0.343874097037	-2.712595212092	0.229094692789
H	1.159381837870	-2.900845740436	0.915702618937
C	0.215072989680	2.755430981643	4.072256055601
H	0.379686179979	3.770484409934	3.727296788424
C	-0.415948582468	3.516823251484	-3.667435207090
C	-0.072258970000	-3.643477538317	-0.688020445202
C	0.393801051682	1.311633754778	-0.723334582760
C	-0.387294166867	-1.487003917957	0.171804808179
C	0.507440257120	-4.975534494128	-0.870430817212
C	-1.394259765212	-1.518099510116	-0.782519649575
C	1.400093327519	0.781237602657	-1.518627808513
C	2.418552599576	-0.266531856114	-1.183648951619
H	3.382182580844	-0.046356380280	-1.652939363019
H	2.099280584746	-1.257969326936	-1.525766287648
H	2.570256317721	-0.321937160760	-0.102294906746
C	-2.415496532978	-0.472977225852	-1.117906429379

H	-3.377664888694	-0.927004379839	-1.373861381056
H	-2.096932489738	0.137913948100	-1.970759205696
H	-2.570219534230	0.198120493863	-0.268553360281
H	0.330757650937	-6.547621352687	-1.901068728455
H	-1.712728714263	4.856815763972	-3.922821466037

Table S13. Optimized B3LYP/6-31G* Cartesian coordinates (in Å) for C_1 conformer of compound 3b in dichloromethane.

E = -2094.512570 a.u.

S	0.118180476312	2.080204038659	-2.658854494884
S	-0.113695571738	-1.497618488862	-3.026622055772
O	0.052887369787	-4.243992495518	-4.007985990913
O	-0.309967471800	2.445989947662	2.623503897995
C	-0.002921947694	0.626110130001	1.104885225569
O	-0.056772902639	5.063046055346	-3.002382190311
O	-0.024477205748	5.580678697269	-0.799764319577
C	-0.083150849028	0.329202476021	3.632741128295
C	-0.153038928976	1.234322925930	2.454024838688
C	-0.004788434385	-0.839039310749	0.954191263775
C	0.078173712280	-1.061240003447	3.489741528706
C	0.161937831784	-1.870353039076	4.631906602051
H	0.284133449047	-2.939689424086	4.499758447680
C	0.099084319837	2.851112354905	-0.119455767442
H	0.038081268476	3.501332790498	0.739438110639
C	0.075189691229	3.307329348750	-1.403338379438
C	-0.078610918734	0.079062479066	6.042961211949
H	-0.138994545436	0.520428879767	7.033441778415
O	0.040940451703	-5.342119730208	-2.029813690061
C	0.084908719021	-1.305370192368	5.900562116787
H	0.149945608452	-1.938526936867	6.780550184967
C	0.142748838282	-1.708770419194	2.151477709771
O	0.294887404447	-2.930046885868	2.071758296091
C	-0.098131537407	-2.766744119415	-0.698849734100
H	-0.038218640517	-3.584267229351	0.003049329712
C	-0.161558872095	0.889656470778	4.915763825070
H	-0.284123817531	1.963432376008	5.004127813047
C	-0.006757459183	4.720106152952	-1.835455302098
C	-0.070766833221	-2.957119169751	-2.047991161755
C	0.150187856701	1.423298930117	-0.011602975914
C	-0.152994329528	-1.392347740815	-0.302127098558
C	0.010618676983	-4.304566374379	-2.662106063215
C	-0.452441301961	-0.471540262003	-1.492655062536
C	0.452590998287	0.765175612777	-1.364857438139
C	1.971427947898	0.451293741883	-1.427475212344
H	2.244999393923	0.033759157356	-2.398452476691
H	2.251205177696	-0.261612504666	-0.646838853581
H	2.537664896243	1.372717876986	-1.270954947434
C	-1.971302970906	-0.152376106200	-1.495432925170

H	-2.242833610697	0.452386675694	-2.362850385421
H	-2.253751207688	0.388325328465	-0.587822100832
H	-2.537058204909	-1.086697851760	-1.529076534399
H	0.108363574204	-5.159256721268	-4.344131607704
H	-0.087178523830	6.481013488935	-1.172725176193

Table S14. Optimized broken-symmetry B3LYP/6-31G* Cartesian coordinates (in Å) for transition state between 3a and 3b in dichloromethane.

E = -2094.463410 a.u.
$\langle S^2 \rangle$ = 0.864338
ν^{TV} = 739*i* cm^{-1}

E(triplet) = -2094.458489 a.u.
$\langle S^2 \rangle$ = 2.028949

S	2.100049031681	2.579340077903	-0.645686722263
S	-1.488718254550	2.978935251711	0.645822141627
O	2.281973596072	-2.608634884364	-0.832139911201
C	1.122218992890	-2.480165252839	-0.437789743252
C	0.538511691159	-1.129119223578	-0.204903955894
O	-2.800858451225	-2.040458823174	0.855682024661
C	-0.779165951330	-0.981963497650	0.212757941294
C	0.785352793131	-4.949273444021	-0.421488708091
H	1.816864390011	-5.044351917213	-0.742868051266
C	-1.343385597667	-5.924606667465	0.190558508464
H	-1.965743260898	-6.801045282383	0.344931847619
C	-2.888133363568	2.091715487858	0.090912639993
C	-1.073318124069	-3.520246426855	0.205766969264
C	-2.654345971197	0.722375579216	0.047326070208
H	-3.411156420496	0.028243563408	-0.290856215679
C	-0.014410769036	-6.073087354480	-0.221918127013
H	0.395666420259	-7.064881774785	-0.387749953333
C	-1.645914834537	-2.170921982376	0.448507518579
C	3.267828626368	1.408285100957	-0.087102818455
C	0.261319960923	-3.669331255467	-0.209314147634
C	-1.355591373772	0.348602169897	0.410380122268
C	-1.871064215420	-4.652476594124	0.404347199735
H	-2.898186799165	-4.517646439396	0.725489435106
C	-0.503426279610	1.483100359460	0.766319017431
C	2.743258788218	0.122253100898	-0.038337218934
H	3.322551325084	-0.724706823504	0.301539077792
C	1.393386164265	0.041869949421	-0.403634732944
C	0.350991737430	1.448248245378	2.025019751124
H	0.946246369130	0.535118404757	2.073474812103
H	-0.299417090045	1.474550699113	2.904968450733
H	1.023382430416	2.309624723661	2.072913209551
C	0.812584561815	1.335243808284	-0.763478706844
C	-0.029872085319	1.484816063779	-2.021824352214
H	0.609622943508	1.362954981666	-2.901725158591
H	-0.494340255826	2.473684749456	-2.073571498463
H	-0.812735891727	0.726153155129	-2.066820883247
C	4.598976719295	1.862516883225	0.303467471485
O	4.963525284956	3.028607297201	0.278309785075
O	5.401958322412	0.845334390328	0.696155911482

C	-4.137022169182	2.731282408867	-0.317431911392
O	-5.130759046316	2.128900657167	-0.690812102398
O	-4.078927924189	4.082294107452	-0.230452095972
H	6.264049684809	1.240499573118	0.926472343905
H	-4.946108308528	4.426789250565	-0.516013738064

Table S15. Optimized B3LYP/6-31G* Cartesian coordinates (in Å) for C_2 conformer of compound 4a in dichloromethane.

E = -6859.574239 a.u.

S	-1.497568714460	-2.347203207385	-2.257591836307
S	1.497568714460	2.347203210690	-2.257591832871
O	-0.539058260660	-2.625814627823	2.430278666671
C	-0.294947978241	-1.422556526851	2.441963629944
C	-0.166447382850	-0.662636946175	1.149753717450
O	0.539058260660	2.625814624266	2.430278670514
C	0.166447382850	0.662636944493	1.149753718420
C	-0.265936050682	-1.374111809523	4.936206562277
H	-0.474838375828	-2.438216014437	4.916538787320
C	0.132573559928	0.687131364984	6.141537482333
H	0.236096644628	1.220216504604	7.081931444706
C	0.187018060357	3.241077244521	-1.539861671086
C	0.134167134787	0.689156548834	3.723365512207
C	-0.257950527149	2.669793882790	-0.389354954639
H	-1.057530018957	3.078222611889	0.214612249269
C	-0.132573559928	-0.687131373973	6.141537481327
H	-0.236096644628	-1.220216514969	7.081931442920
C	0.294947978241	1.422556523277	2.441963632026
C	-0.187018060357	-3.241077242267	-1.539861675830
C	-0.134167134787	-0.689156554284	3.723365511199
C	0.446572037148	1.452958795025	-0.072471731532
C	0.265936050682	1.374111802298	4.936206564288
H	0.474838375828	2.438216007241	4.916538790889
C	1.439280315964	1.151312194668	-0.982303632846
C	0.257950527149	-2.669793882220	-0.389354958546
H	1.057530018957	-3.078222612203	0.214612244764
C	-0.446572037148	-1.452958794919	-0.072471733659
C	2.425601174477	0.021692767963	-0.989621651320
H	3.412082080536	0.358046414359	-1.324906617987
H	2.105528206325	-0.790926154578	-1.652308751950
H	2.534052121011	-0.394084050486	0.015877176512
C	-1.439280315964	-1.151312193230	-0.982303634531
C	-2.425601174477	-0.021692766514	-0.989621651351
H	-3.412082080536	-0.358046412420	-1.324906618511
H	-2.105528206325	0.790926156997	-1.652308750792
H	-2.534052121011	0.394084050463	0.015877177088
Br	0.454944091130	-4.818685304992	-2.353438079869
Br	-0.454944091130	4.818685308437	-2.353438072816

Table S16. Optimized B3LYP/6-31G* Cartesian coordinates (in Å) for C_2 conformer of compound 4b in dichloromethane.

E = -6859.569829 a.u.

S	-0.118113271470	-1.792830775903	-2.650270712158
S	0.118113271470	1.792830775409	-2.650270712492
O	0.264023662725	-2.709573998465	2.570611370122
C	0.133992343115	-1.481540181481	2.519977095243
C	-0.001256054041	-0.738608580735	1.245487711496
O	-0.264023662725	2.709573998945	2.570611369616
C	0.001256054041	0.738608580968	1.245487711358
C	0.152540731239	-1.387664124291	5.005352467728
H	0.267350499947	-2.465687157593	4.982831243384
C	-0.077285432711	0.696455441412	6.209704746505
H	-0.136564615587	1.237109394945	7.149890520869
C	0.063126740497	3.132369801002	-1.523840875574
C	-0.076007769854	0.699370997897	3.785686896203
C	0.087534607372	2.829943332853	-0.200211973108
H	0.020340385236	3.567391977065	0.584223446235
C	0.077285432711	-0.696455440253	6.209704746635
H	0.136564615587	-1.237109393610	7.149890521100
C	-0.133992343115	1.481540181951	2.519977094966
C	-0.063126740497	-3.132369801287	-1.523840874989
C	0.076007769854	-0.699370997190	3.785686896334
C	0.145697711701	1.420163364396	0.053900269510
C	-0.152540731239	1.387664125225	5.005352467469
H	-0.267350499947	2.465687158523	4.982831242924
C	0.454650387715	0.620561330177	-1.221256320041
C	-0.087534607372	-2.829943332890	-0.200211972579
H	-0.020340385236	-3.567391976955	0.584223446901
C	-0.145697711701	-1.420163364385	0.053900269775
C	1.971031546520	0.300669610034	-1.249390900129
H	2.246514032722	-0.217938793153	-2.169966482761
H	2.245928109614	-0.326359098995	-0.396892534001
H	2.538337667705	1.232609537703	-1.189088719251
C	-0.454650387715	-0.620561330405	-1.221256319925
C	-1.971031546520	-0.300669610267	-1.249390900073
H	-2.246514032722	0.217938792748	-2.169966482802
H	-2.245928109614	0.326359098921	-0.396892534062
H	-2.538337667705	-1.232609537925	-1.189088719021
Br	0.040315268796	-4.878437677080	-2.218252599855
Br	-0.040315268796	4.878437676666	-2.218252600766

Table S17. Optimized broken-symmetry B3LYP/6-31G* Cartesian coordinates (in Å) for transition state between 4a and 4b in dichloromethane.

S	1.823390478478	2.556016058565	-0.713316663014
S	-1.822052190290	2.556963084356	0.713534020983
O	2.595345066035	-2.574501632191	-0.744185875249
C	1.411557181921	-2.560478371019	-0.389258818437
C	0.675689799722	-1.287661121300	-0.200058748488
O	-2.596741358200	-2.573134897181	0.744227248949
C	-0.676405586378	-1.287306597014	0.200115551072
C	1.351026825636	-5.052039814791	-0.353647030158
H	2.399451620927	-5.031197464617	-0.630566688607
C	-0.679145865961	-6.256977340275	0.176417455988
H	-1.205457021957	-7.197076494212	0.313836951490
C	-3.105112994726	1.512277352029	0.157755924423
C	-0.680809560363	-3.835993049774	0.178000822408
C	-2.743340585306	0.191235648991	0.074156141752
H	-3.410435656976	-0.581852890719	-0.278376447653
C	0.675694995400	-6.257334073252	-0.176800192432
H	1.201484634909	-7.197710216247	-0.314320977570
C	-1.412959630821	-2.559735467128	0.389255221992
C	3.105892122172	1.510626107781	-0.157573590425
C	0.678699455329	-3.836350944870	-0.178130516674
C	-1.388658221195	-0.039504954537	0.423418588360
C	-1.353809857680	-5.051327648173	0.353391486916
H	-2.402223985256	-5.029934574521	0.630309212924
C	-0.678135465089	1.173809631561	0.802211637257
C	2.743415973809	0.189774081057	-0.074025764655
H	3.410098279238	-0.583683649491	0.278477480343
C	1.388611834934	-0.040231262559	-0.423302101118
C	0.216508783869	1.229336845256	2.027497242644
H	0.895334257440	0.375833664114	2.057557935966
H	-0.398642252293	1.201816026174	2.933140463794
H	0.808779397662	2.149338239287	2.047421750313
C	0.678737045071	1.173476841343	-0.802049049470
C	-0.215877137735	1.229530037396	-2.027332542288
H	0.399258781628	1.201709706105	-2.932976873364
H	-0.807649138178	2.149852859950	-2.047223227105
H	-0.895164427117	0.376395237004	-2.057424184779
Br	4.766514817241	2.233058803061	0.346645814539
Br	-4.765353654922	2.235611320580	-0.346429931631

E = -6859.507392 a.u.
$\langle S^2 \rangle$ = 1.0133
ν^{TV} = 731*i* cm^{-1}

E(triplet) = -6859.505994 a.u.
$\langle S^2 \rangle$ = 2.021735

References

1. Patel, D. G.; Mitchell, T. B.; Myers, S. D.; Carter, D. A.; Novak, F. A. A Suzuki Approach to Quinone-Based Diarylethene Photochromes. *J. Org. Chem.* **2020,** *85* (4), 2646-2653.
2. Spek, A. Structure validation in chemical crystallography. *Acta Crystallogr. Sect. D. Biol. Crystallogr.* **2009,** *65* (2), 148-155.
