# Supplementary figures and images for: Computational and Crystallographic Examination of Naphthoquinone Based Diarylethene Photochromes
Source: Molecules. 2020 Jun 5;25(11):2630. doi: 10.3390/molecules25112630 (PMC7321381; doi:10.3390/molecules25112630)

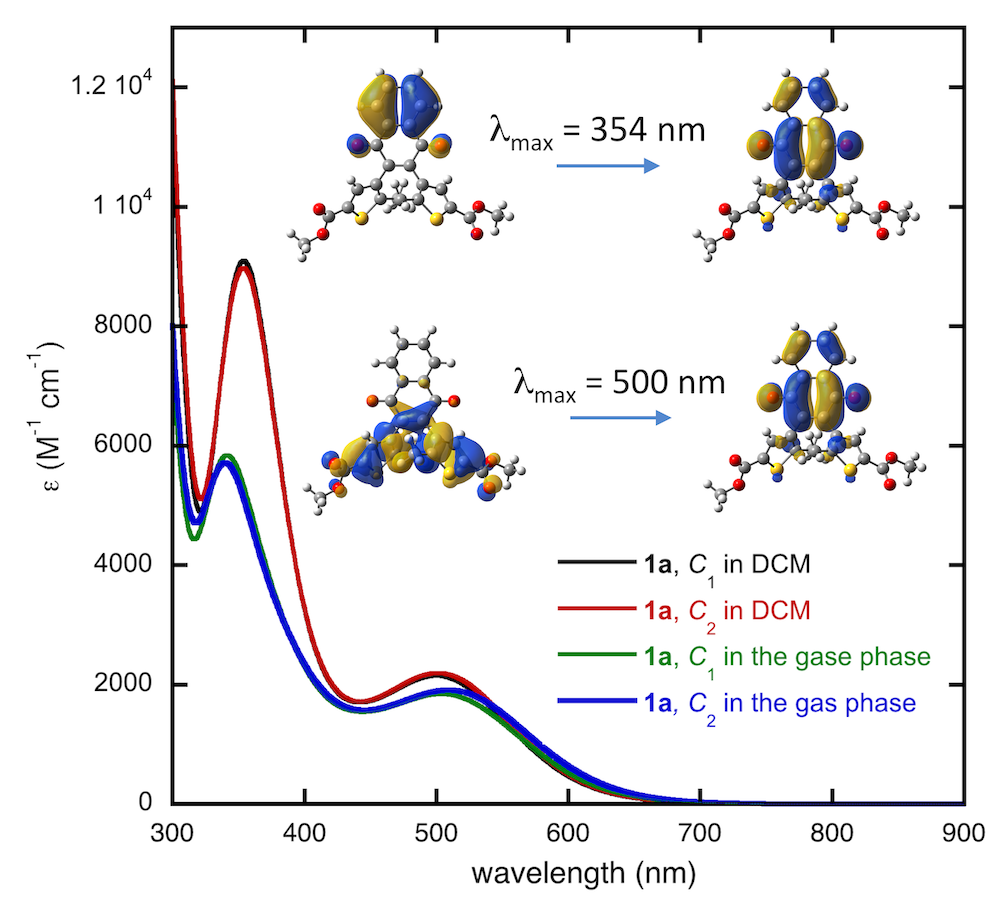

Supplement: Supplementary file 1 [file molecules-25-02630-s001.zip › Supporting Information and Figures/Supporting Information Figures and Videos/Fig S1 - open B3LYP.tiff]

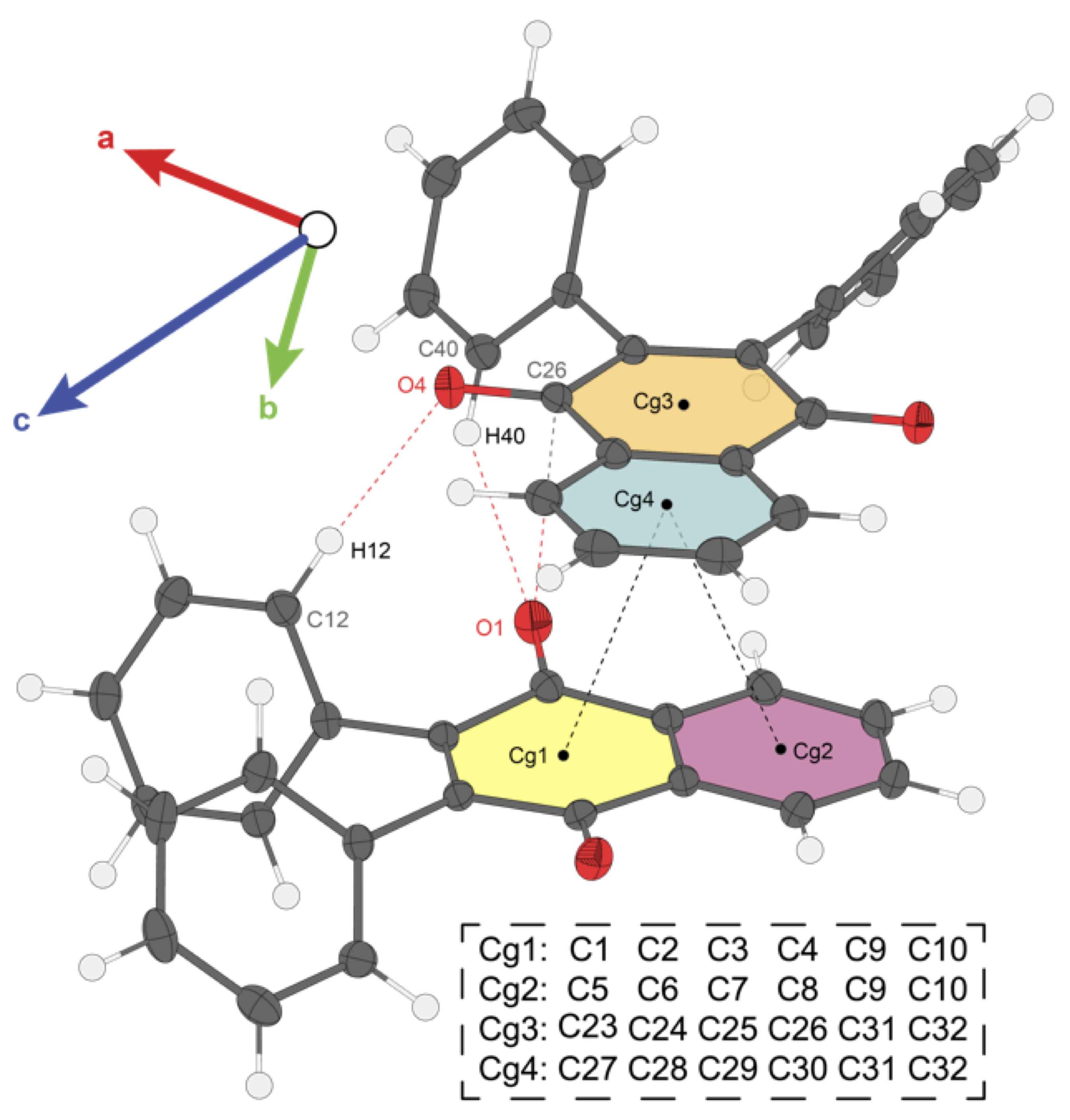

Supplement: Supplementary file 1 [file molecules-25-02630-s001.zip › Supporting Information and Figures/Supporting Information Figures and Videos/Figure S5.tiff]

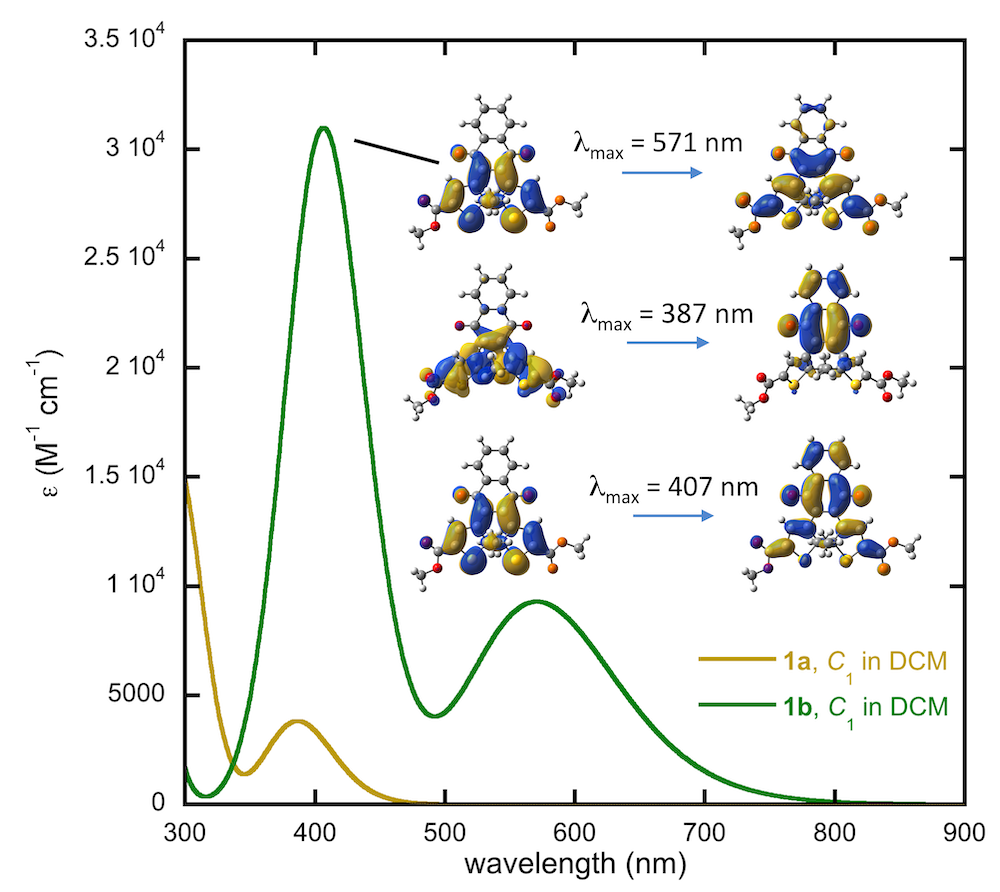

Supplement: Supplementary file 1 [file molecules-25-02630-s001.zip › Supporting Information and Figures/Supporting Information Figures and Videos/Figure S3 CAM.tiff]

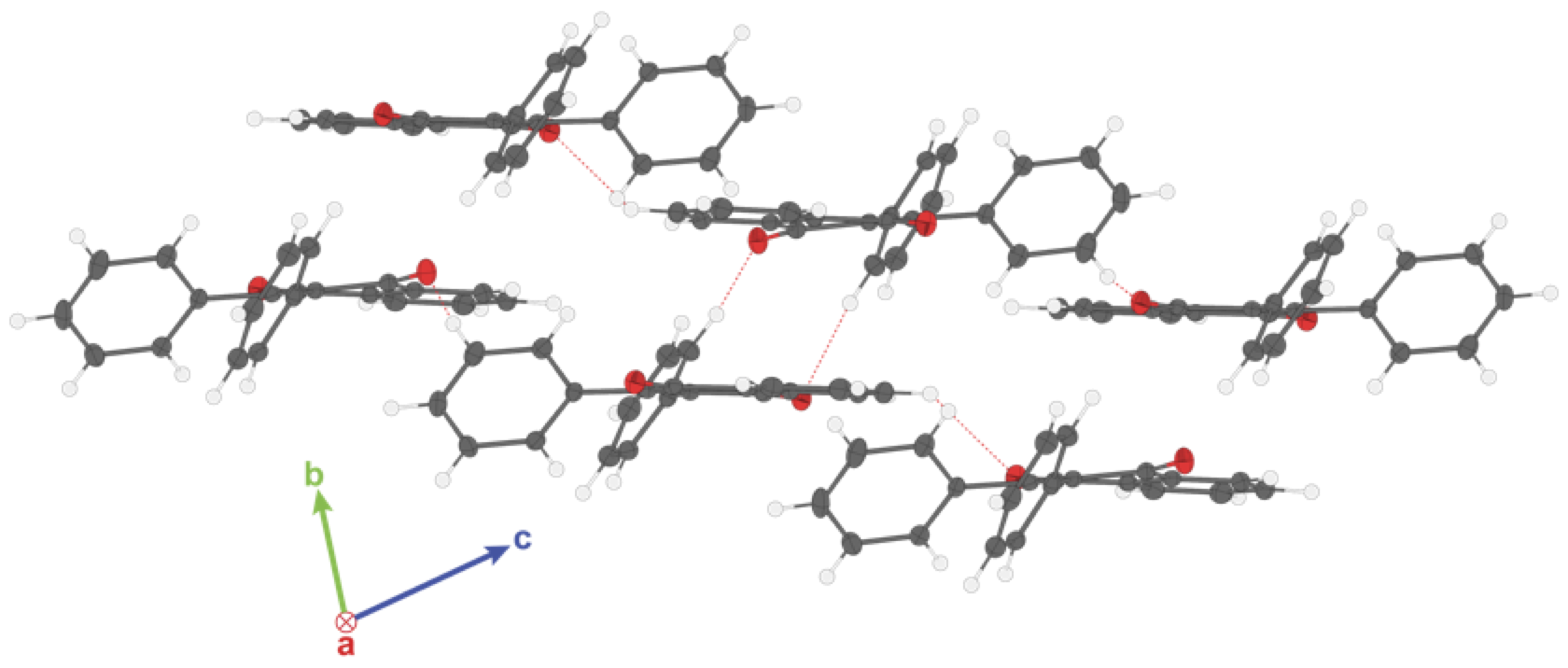

Supplement: Supplementary file 1 [file molecules-25-02630-s001.zip › Supporting Information and Figures/Supporting Information Figures and Videos/Figure S7.tiff]

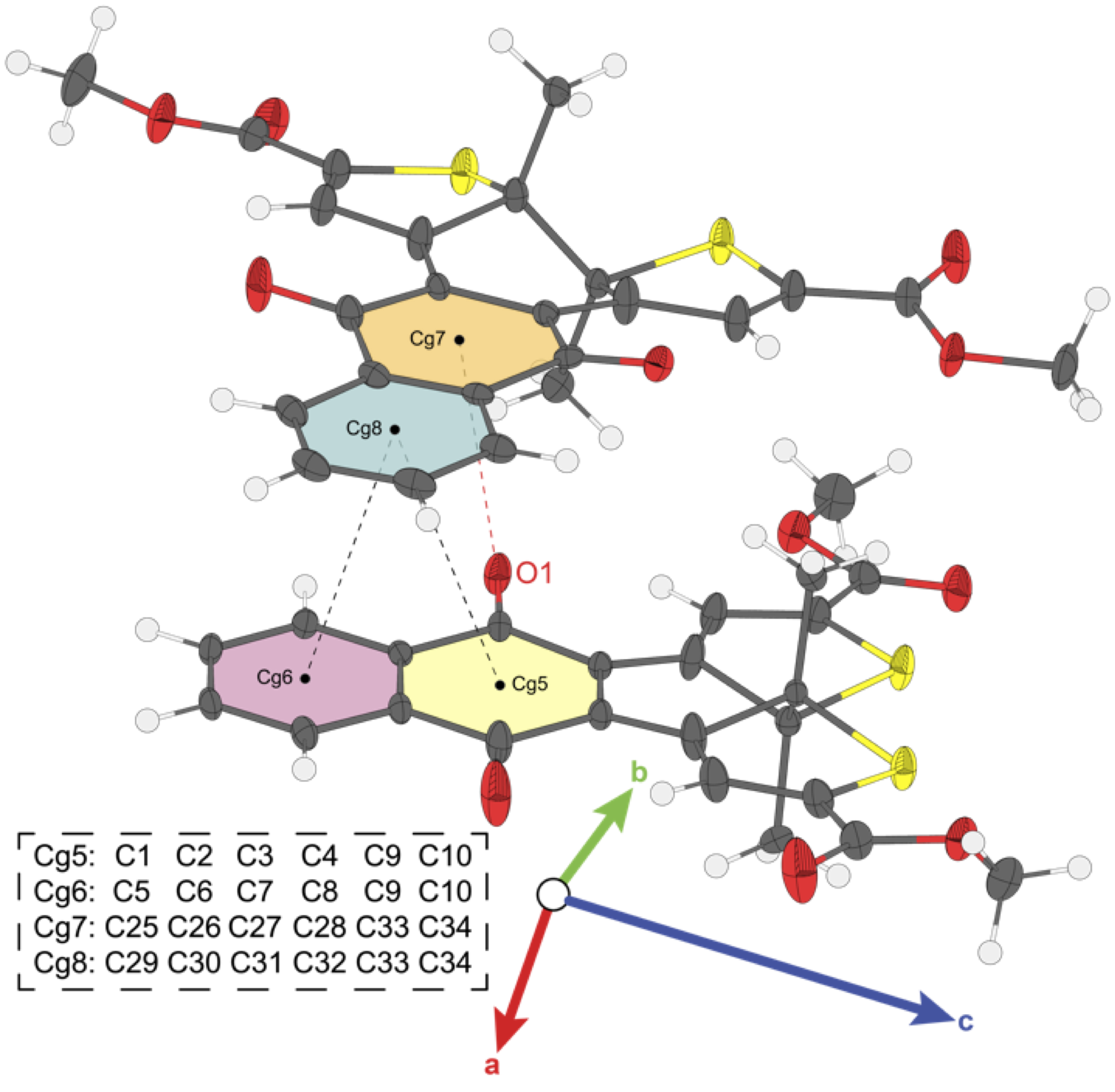

Supplement: Supplementary file 1 [file molecules-25-02630-s001.zip › Supporting Information and Figures/Supporting Information Figures and Videos/Figure S6.tiff]

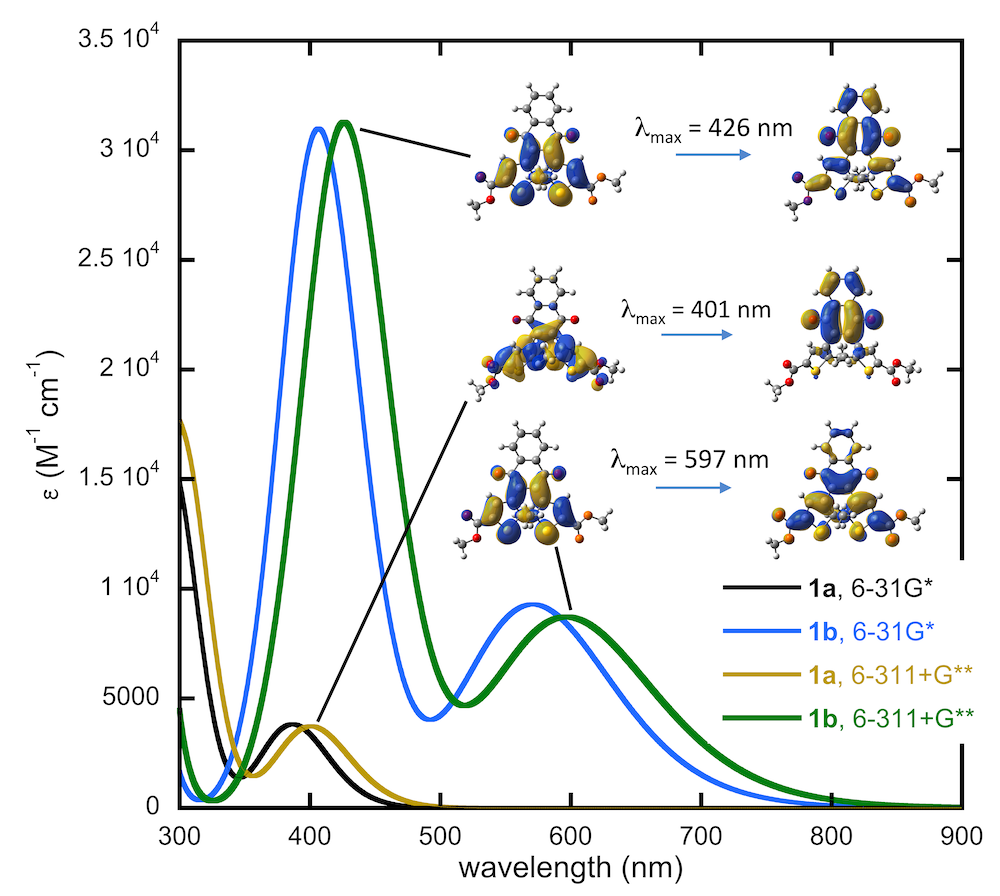

Supplement: Supplementary file 1 [file molecules-25-02630-s001.zip › Supporting Information and Figures/Supporting Information Figures and Videos/Figure S4 G* vs G**.tiff]

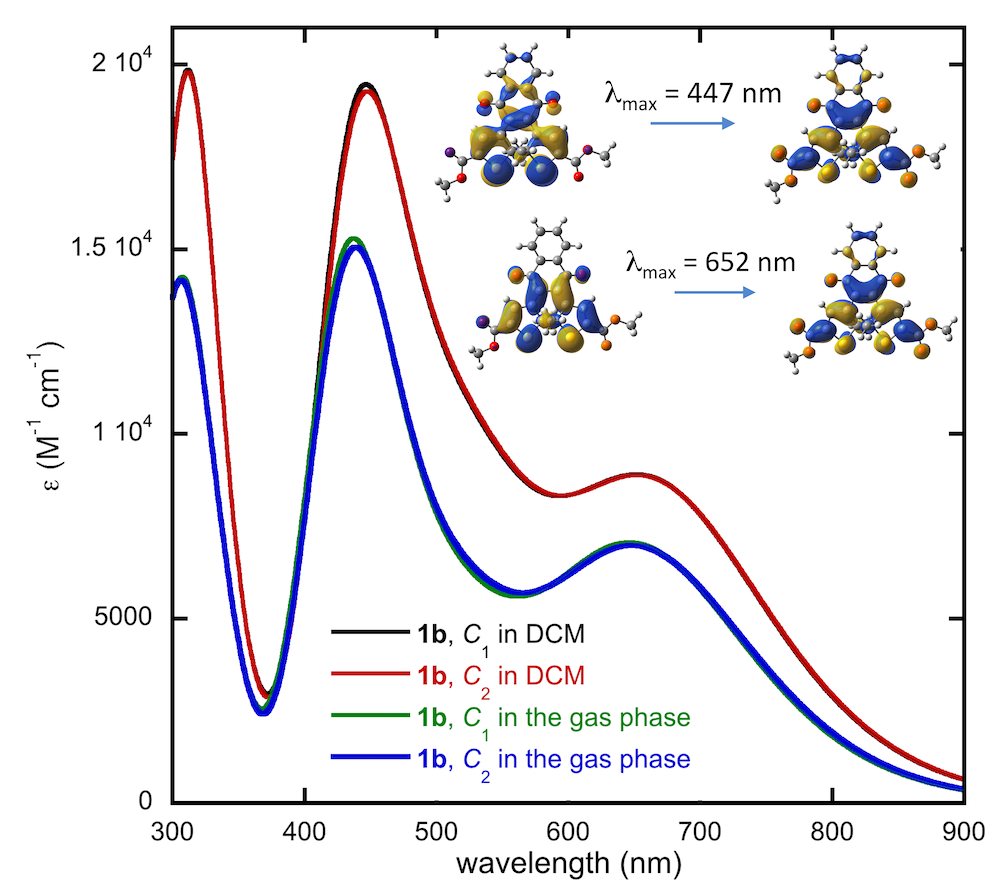

Supplement: Supplementary file 1 [file molecules-25-02630-s001.zip › Supporting Information and Figures/Supporting Information Figures and Videos/Figure S2 closed B3LYP.tiff]

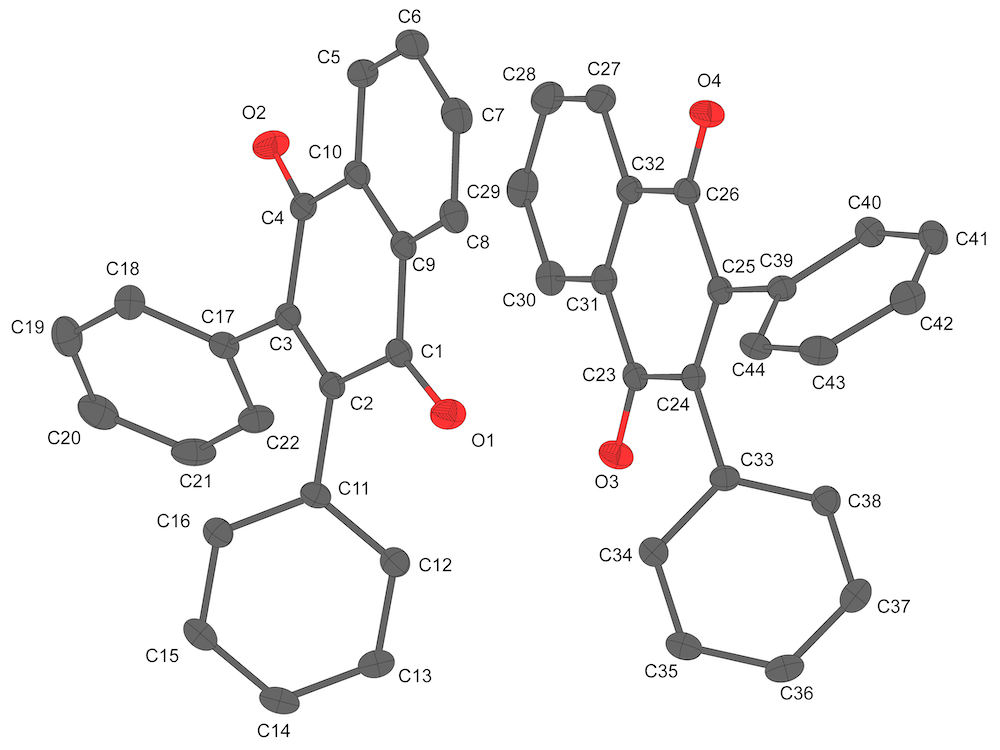

Supplement: Supplementary file 1 [file molecules-25-02630-s001.zip › Supporting Information and Figures/Manuscript Figures/Figure 4.tiff]

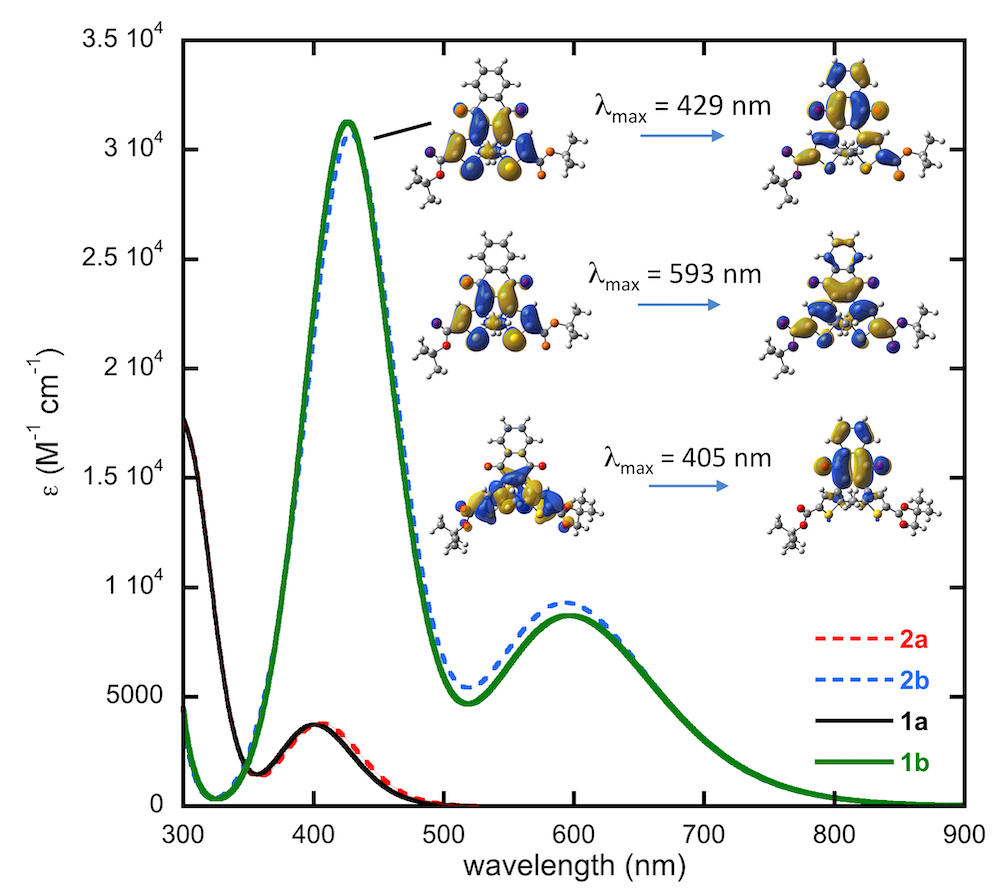

Supplement: Supplementary file 1 [file molecules-25-02630-s001.zip › Supporting Information and Figures/Manuscript Figures/Figure 8.tiff]

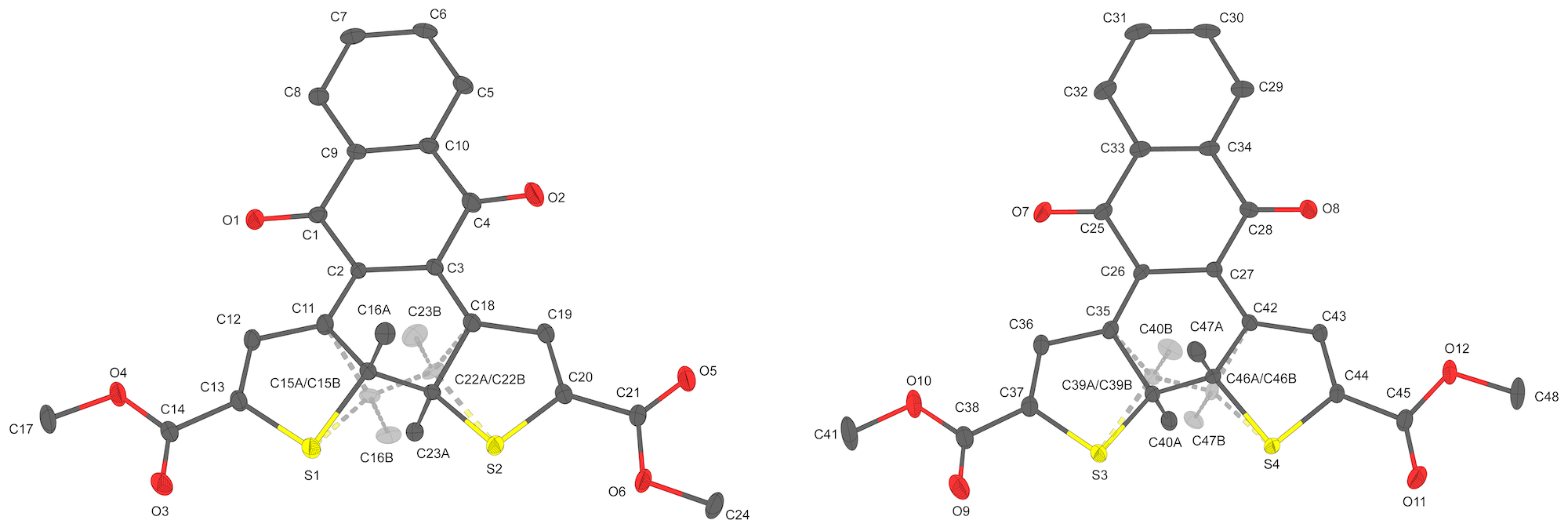

Supplement: Supplementary file 1 [file molecules-25-02630-s001.zip › Supporting Information and Figures/Manuscript Figures/Figure 5.tif]

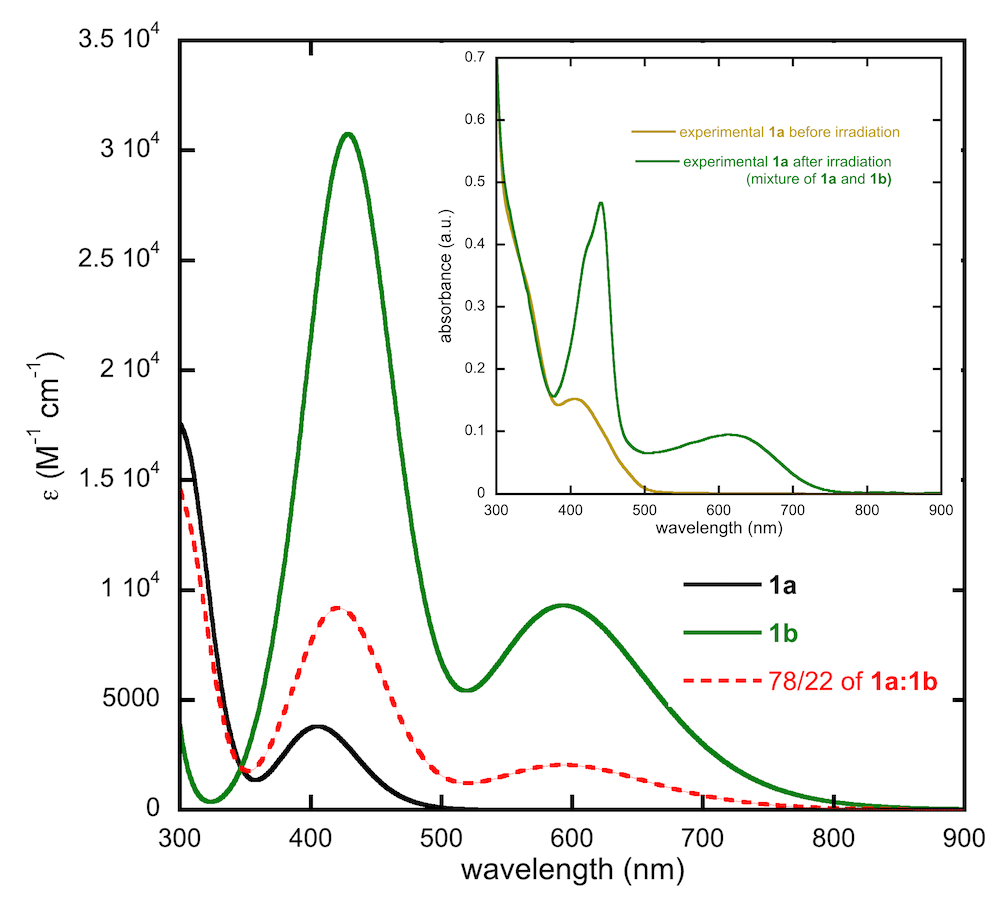

Supplement: Supplementary file 1 [file molecules-25-02630-s001.zip › Supporting Information and Figures/Manuscript Figures/Figure 9.tiff]

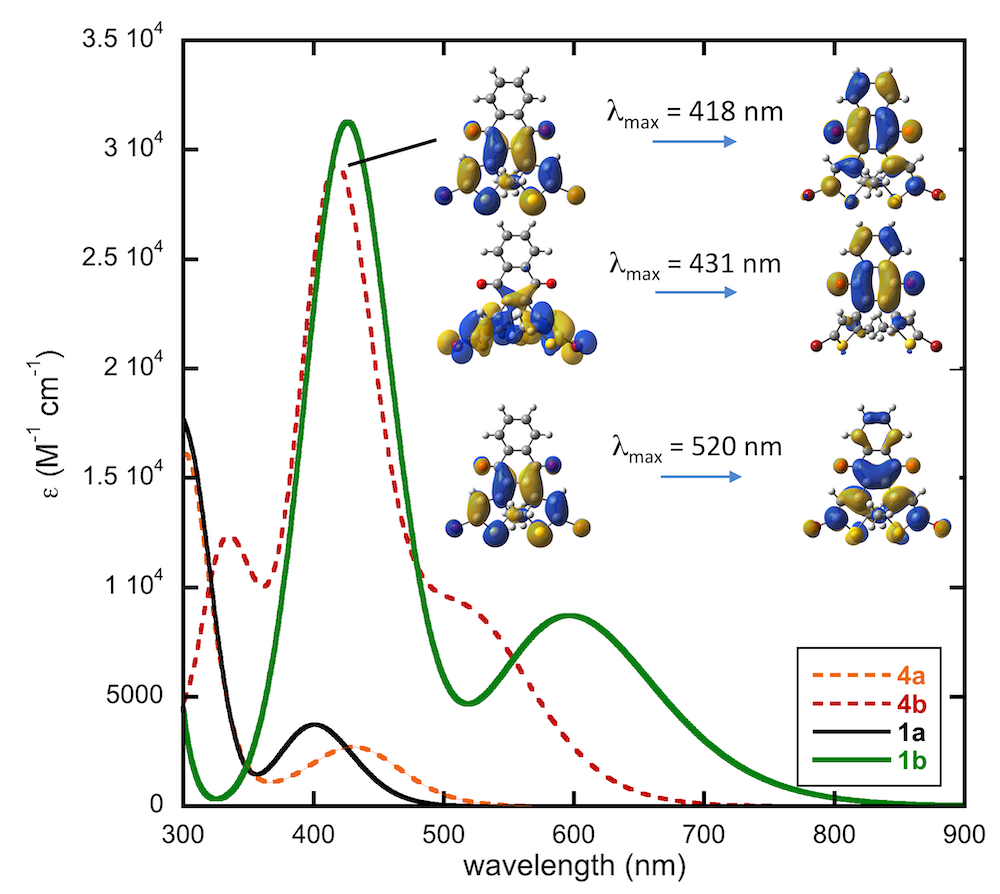

Supplement: Supplementary file 1 [file molecules-25-02630-s001.zip › Supporting Information and Figures/Manuscript Figures/Figure 11 top.tiff]

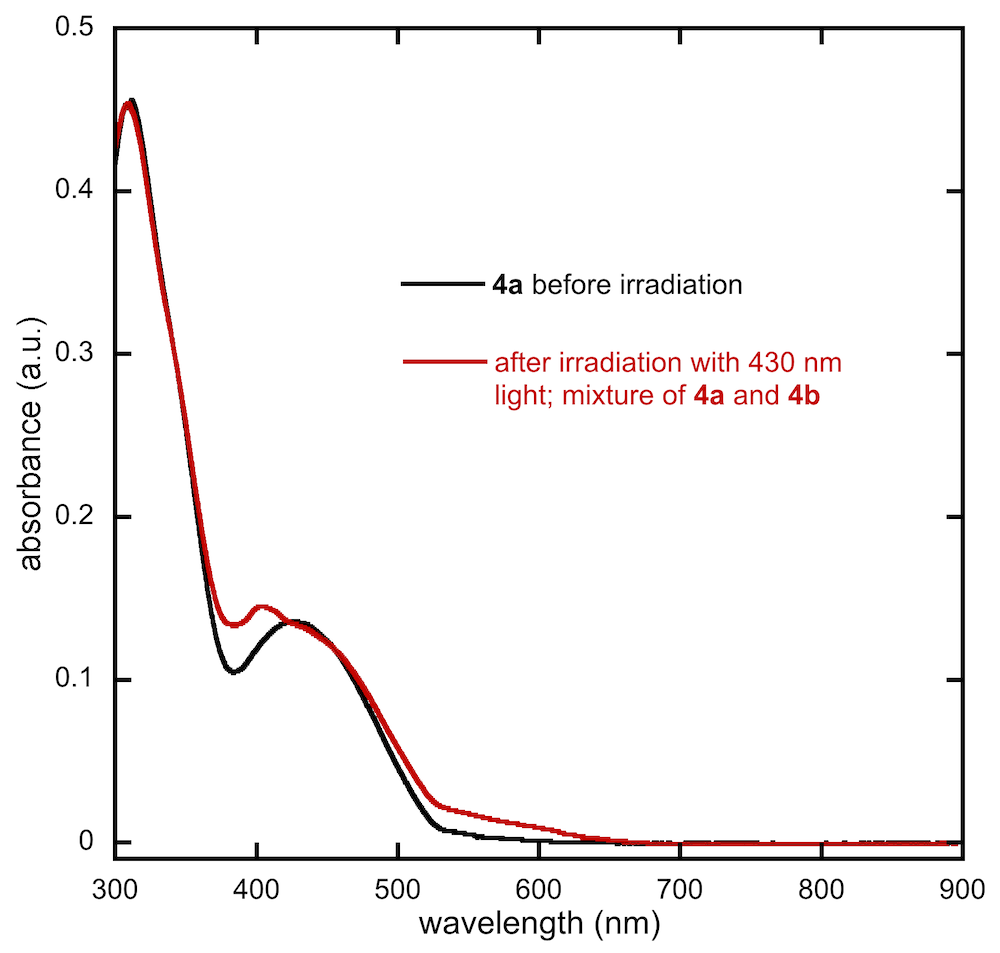

Supplement: Supplementary file 1 [file molecules-25-02630-s001.zip › Supporting Information and Figures/Manuscript Figures/Figure 11 bottom.tiff]

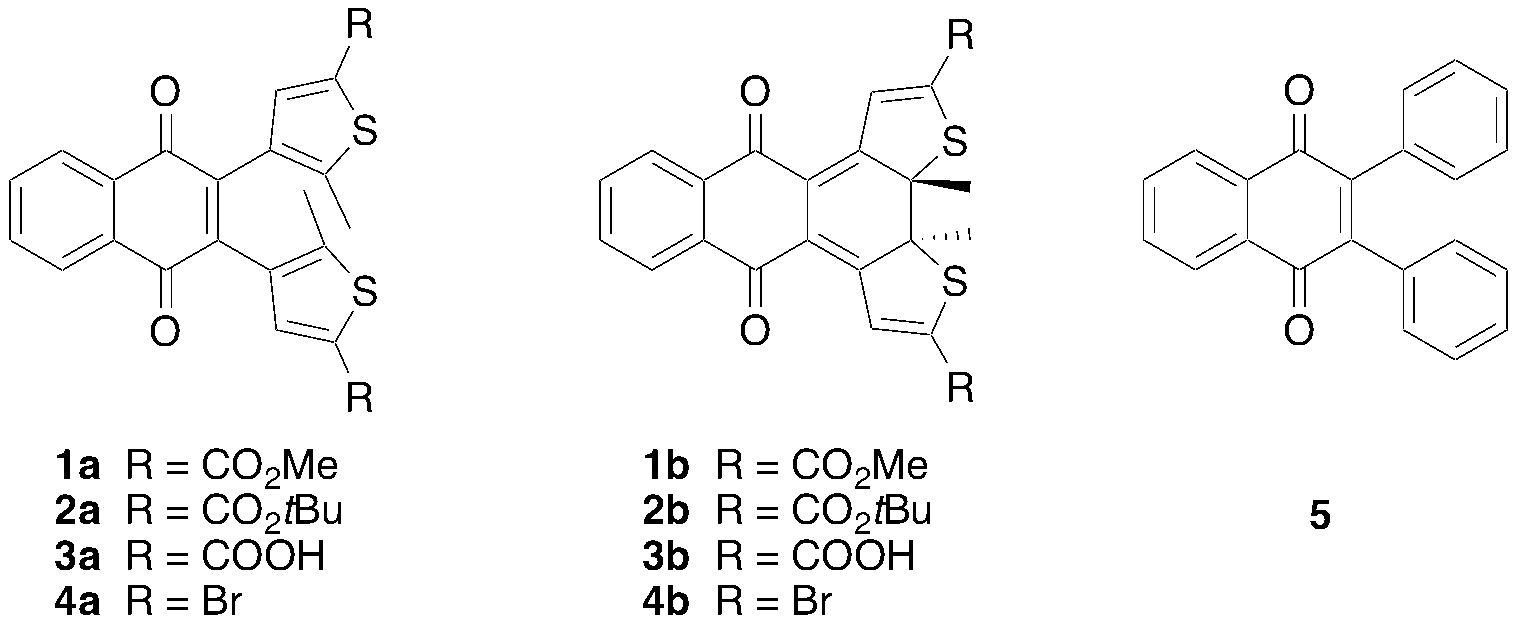

Supplement: Supplementary file 1 [file molecules-25-02630-s001.zip › Supporting Information and Figures/Manuscript Figures/Figure 2.tiff]

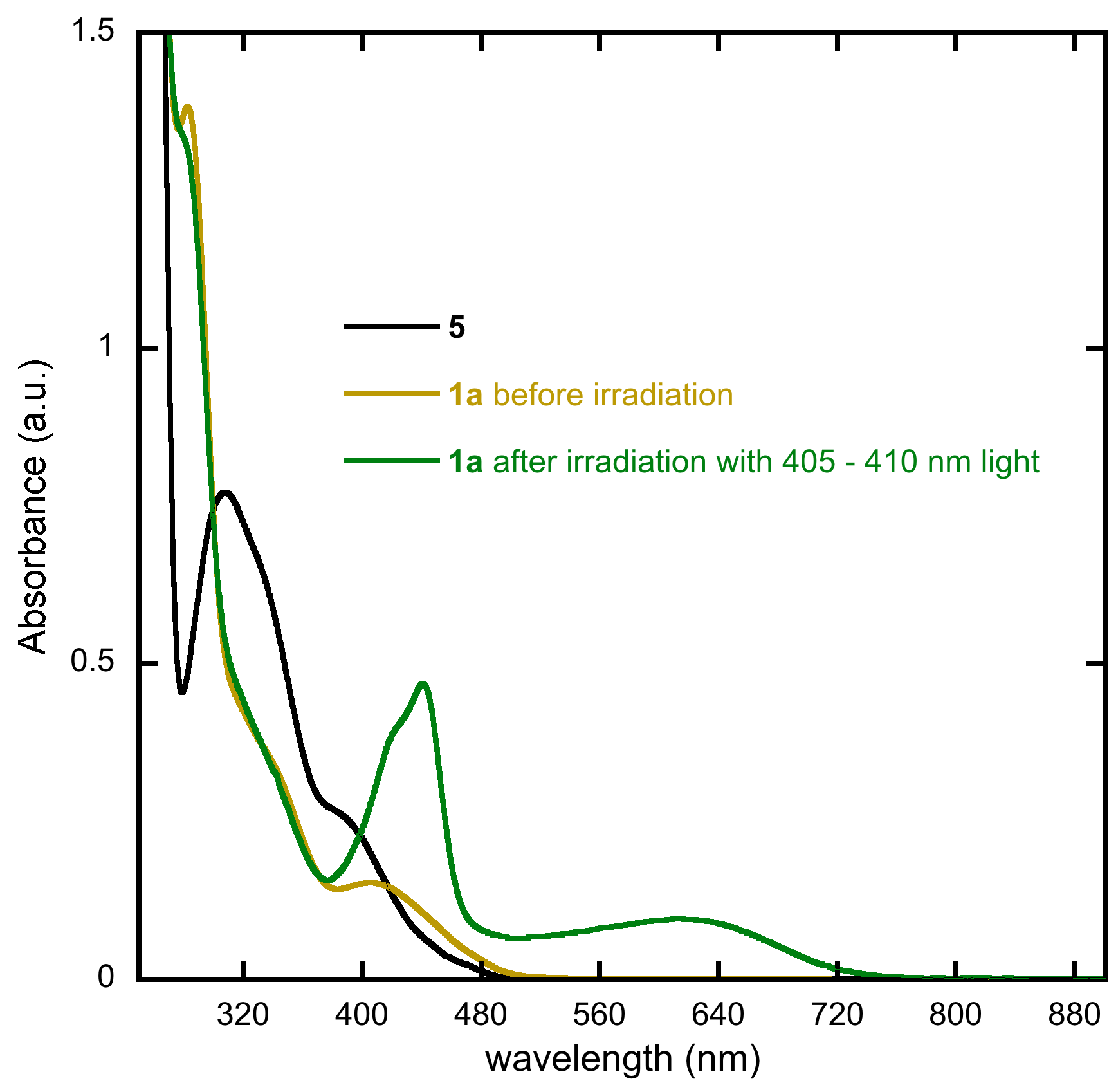

Supplement: Supplementary file 1 [file molecules-25-02630-s001.zip › Supporting Information and Figures/Manuscript Figures/Figure 3.tiff]

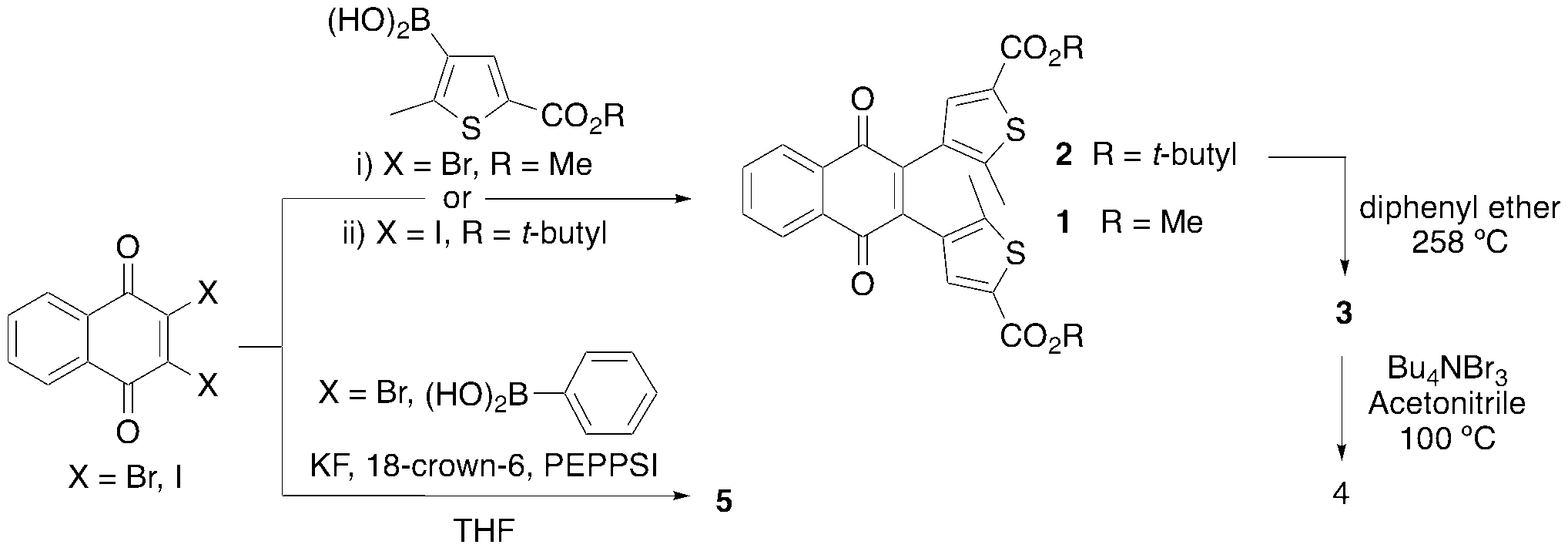

Supplement: Supplementary file 1 [file molecules-25-02630-s001.zip › Supporting Information and Figures/Manuscript Figures/Scheme 1.tiff]

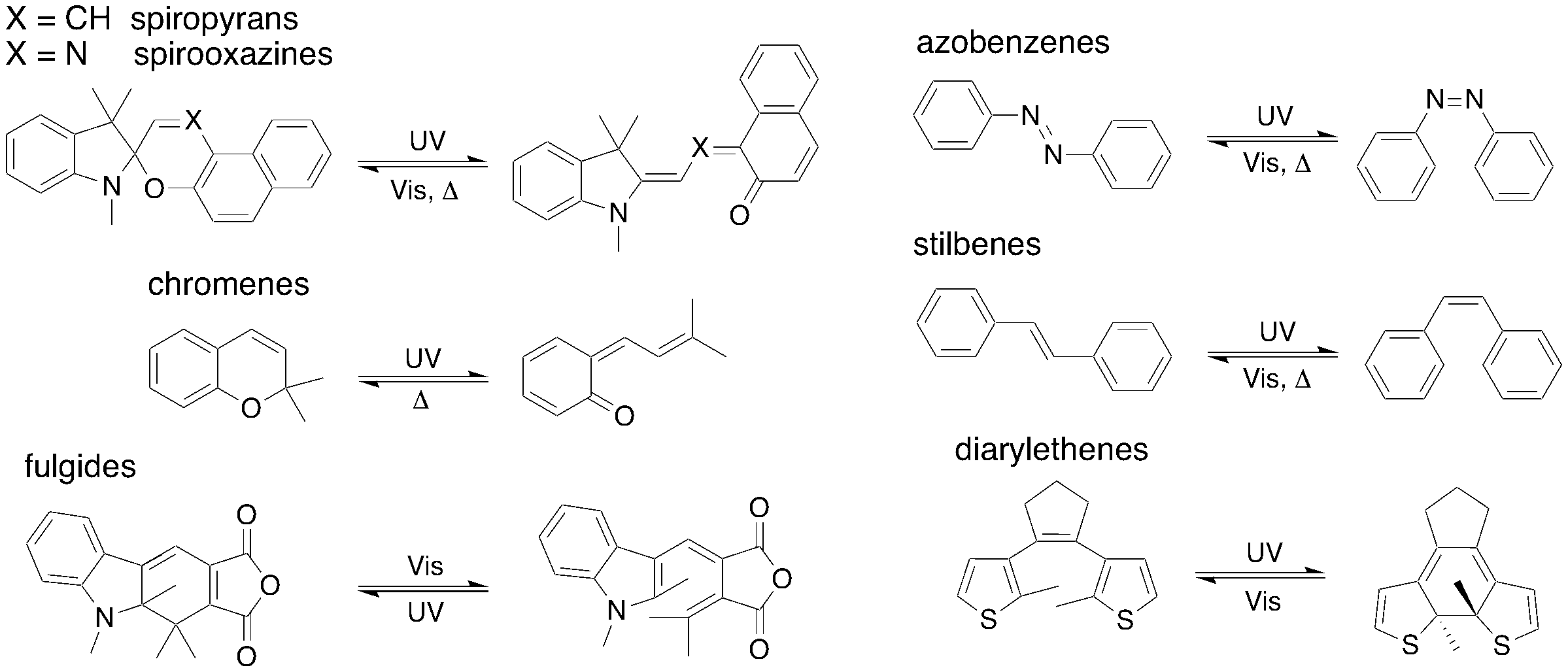

Supplement: Supplementary file 1 [file molecules-25-02630-s001.zip › Supporting Information and Figures/Manuscript Figures/Figure 1.tiff]

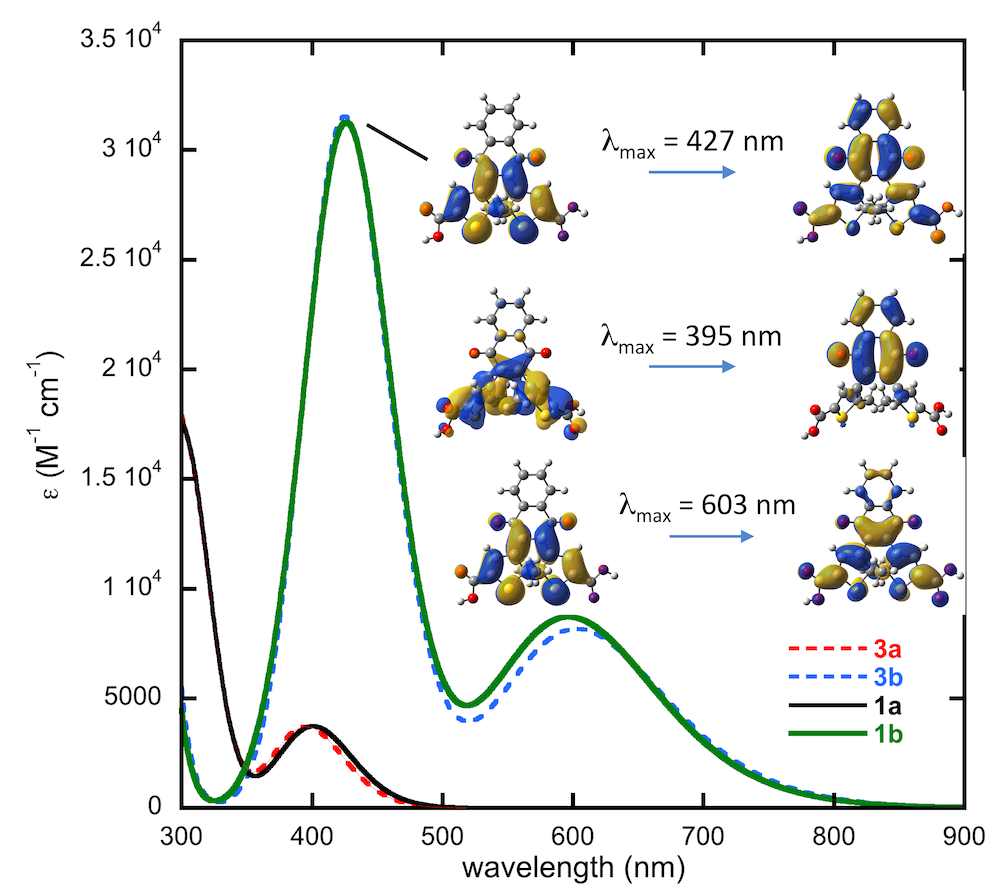

Supplement: Supplementary file 1 [file molecules-25-02630-s001.zip › Supporting Information and Figures/Manuscript Figures/Figure 10.tiff]

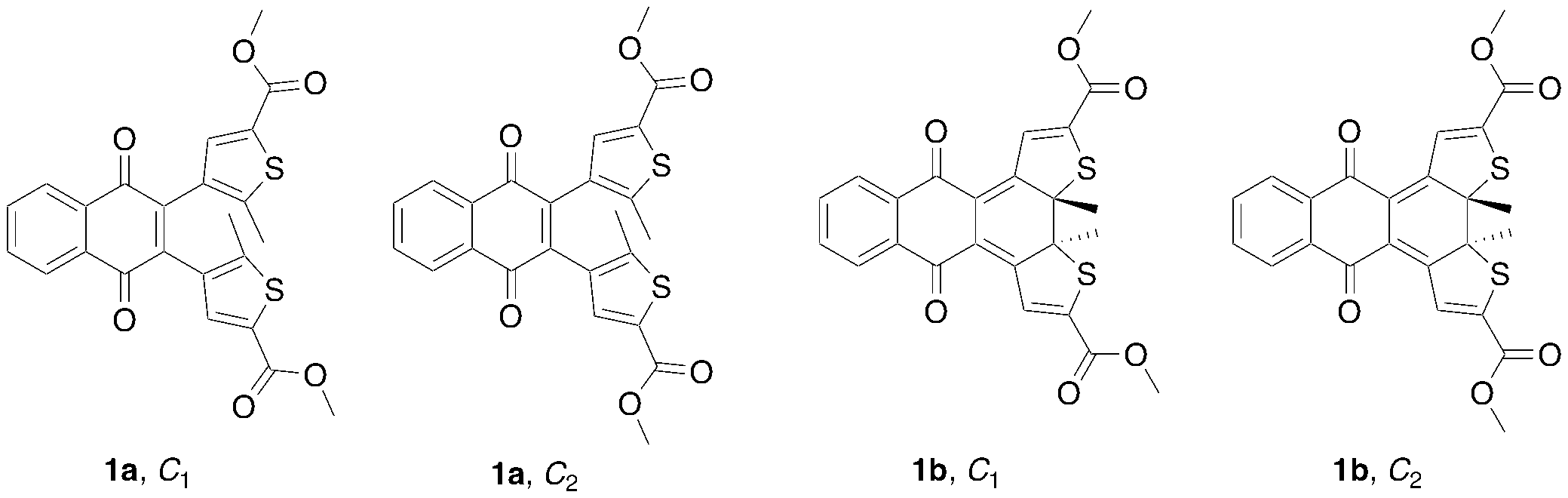

Supplement: Supplementary file 1 [file molecules-25-02630-s001.zip › Supporting Information and Figures/Manuscript Figures/Figure 6.tiff]

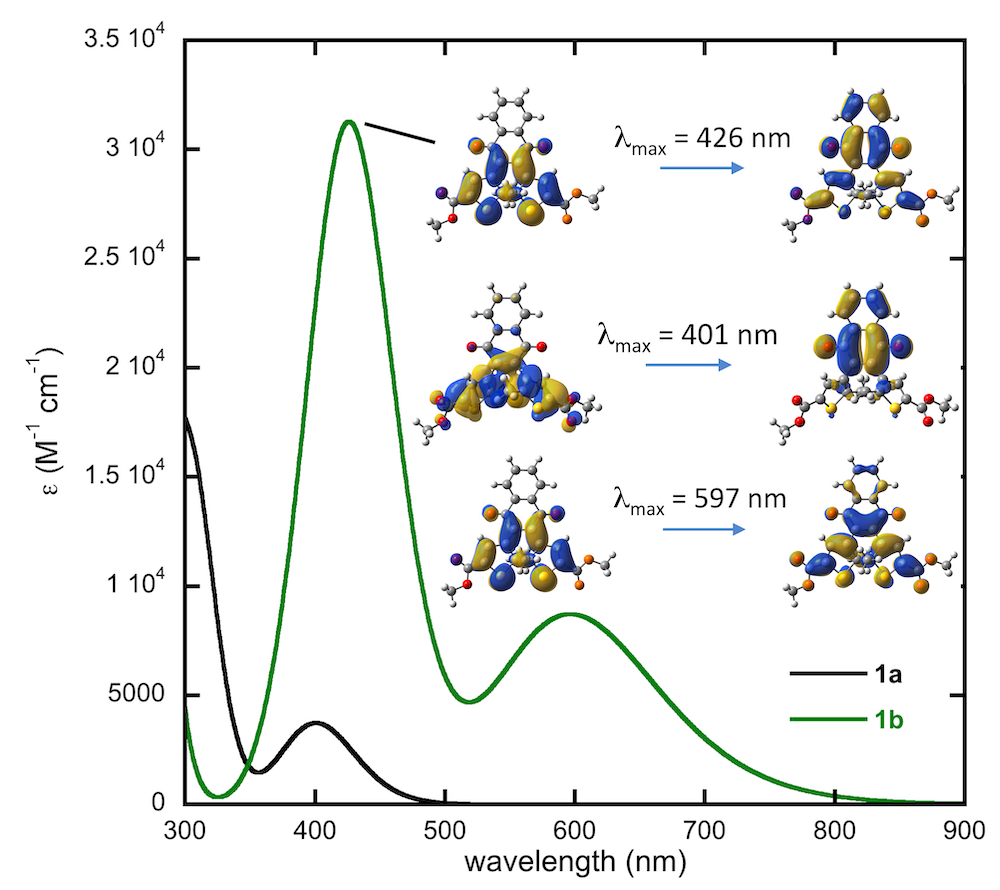

Supplement: Supplementary file 1 [file molecules-25-02630-s001.zip › Supporting Information and Figures/Manuscript Figures/Figure 7.tiff]
